# Prevalence of cardiovascular-kidney-metabolic syndrome in areas of Southern China where ethnic minority populations reside: a cross-sectional study

**DOI:** 10.3389/fendo.2026.1818782

**Published:** 2026-04-22

**Authors:** Kehui Li, Ying Zeng, Manqiu Mo, Yunan Xu, Xiaohua Li, Yuanshan Xu, Zhiqiang Nong, Ling Pan, Rongjie Huang

**Affiliations:** 1 Department of Nephrology, The First Affiliated Hospital of Guangxi Medical University, Nanning, Guangxi, China; 2 Geriatric Department of Endocrinology, The First Affiliated Hospital of Guangxi Medical University, Nanning, Guangxi, China; 3 Department of Medical Research, The First Affiliated Hospital of Guangxi Medical University, Nanning, Guangxi, China; 4 Department of Cardiology, The First Affiliated Hospital of Guangxi Medical University, Nanning, Guangxi, China; 5 Guangxi Key Laboratory of Precision Medicine in Cardio-cerebrovascular Diseases Control and Prevention, Guangxi Medical University, Nanning, Guangxi, China; 6 Guangxi Clinical Research Center for Cardio-cerebrovascular Diseases, Guangxi Medical University, Nanning, Guangxi, China

**Keywords:** associated factors, cardiovascular-kidney-metabolic syndrome, cross-sectional study, multimorbidity, prevalence

## Abstract

**Introduction:**

Epidemiological data and the comorbidity situation of cardiovascular-kidney-metabolic disease (CKM) in ethnic minority regions of southern China are lacking. This study aimed to (1) estimate the prevalence and stage distribution of CKM syndrome and (2) identify factors associated with CKM stages and explore the interrelationships among coexisting chronic conditions in a multi-ethnic population in southern China.

**Methods:**

We analyzed data from the 2020–2021 China Cardiovascular Disease and Risk Factors Surveillance project in Guangxi. CKM syndrome was defined as the coexistence of cardiovascular disease (CVD), chronic kidney disease (CKD), and metabolic disorders. The primary outcome was CKM stage (0–4), and binary CKM variables were used in regression analyses. Prevalence was estimated using data from questionnaires, physical examinations, and laboratory tests. Regression and network analyses were conducted to examine factors associated with CKM and the interrelationships among chronic conditions.

**Results:**

Among 8,552 participants (median age 50 [IQR 36–63]; 41.9% male; 66.6% urban; 42.3% Zhuang), the age- and sex-adjusted prevalence of CKM stages 1–4 was 76.4% (95% CI: 75.5–77.3%), with 17.2% at stage 1, 57.7% at stages 2–3, and 1.4% at stage 4 (all p < 0.001). The prevalence of CKM stages 2–4 was significantly higher among males, older individuals, rural residents, and non-Zhuang populations (p < 0.05). Factors independently associated with CKM (stages 2–4) included male sex, older age, rural residence, lower education level, alcohol consumption, low fruit intake, high red meat intake, and hyperuricemia (all p < 0.05). The prevalence of individual chronic conditions was as follows: hypertension, 39.7%; non-hypertension CVD (other CVD), 1.6%; CKD, 13.8%; obesity, 12.2%; and diabetes, 12.1%. Hypertension was a central condition linking CVD, CKD, hyperuricemia, obesity, and diabetes.

**Conclusion:**

This study reveals a substantial burden of CKM stages 2–3 in Guangxi, China, affecting over half of the population, especially rural elderly men. The findings highlight the need for targeted prevention strategies focusing on modifiable factors associated with CKM in ethnic minority regions.

## 1 Introduction

Cardiovascular disease (CVD), chronic kidney disease (CKD), and metabolic diseases are prevalent chronic noncommunicable diseases that pose major global public health challenges due to their high prevalence, complex interrelationships, associated complications, poor prognosis, and substantial healthcare burden. In 2023, the American Heart Association (AHA) introduced the concept of cardiovascular-renal-metabolic (CKM) syndrome, encompassing CVD, CKD, and metabolic diseases as a unified entity. This classification underscores the intricate interactions among these multimorbidities and their synergistic role in accelerating disease progression. Recent studies have shown that CKM syndrome is highly prevalent worldwide and is a major cause of mortality and disability (1, 2). For instance, an analysis of 10,762 US adults from the NHANES dataset (2011-2020) revealed the following distribution of CKM syndrome:10.6% at stage 0, 25.9% at stage 1, 49.0% at stage 2, 5.4% at stage 3, and 9.2% at stage 4 (3). Notably, the prevalence of each stage remained stable throughout the study period, indicating widespread suboptimal CKM health in the US population. Although global studies such as these have elucidated the high prevalence of CKM syndrome, its epidemiological characteristics may exhibit significant heterogeneity across regions due to variations in population demographics and socioeconomic factors. Systematic research on CKM in southern Chinese populations remains scarce.

Guangxi Zhuang Autonomous Region (Guangxi), located in southern China, is a multi-ethnic settlement with the Zhuang ethnic group as the dominant population, alongside cohabiting Han, Jing, and Yao communities. As one of China’s largest ethnic minority regions, Guangxi has been reported to lag behind the national average in several socioeconomic indicators, including per capita GDP and educational attainment (4). Previous studies have suggested that residents in southern China, particularly in Guangxi, tend to maintain dietary patterns characterized by high intake of vegetables and rice-based staples, along with substantial consumption of pork as the primary source of red meat (5, 6). In addition, disparities in healthcare access and chronic disease management between urban and rural areas have been documented, with rural populations often experiencing lower rates of preventive screening and disease awareness (7). These region-specific socioeconomic and healthcare characteristics may influence the epidemiological profile and burden of CKM syndrome, highlighting the critical need for localized data.

To address this gap, our study investigated the prevalence of CKM stages among adults residing in Guangxi, identified factors associated with CKM, and explored the interrelationships among coexisting chronic conditions within the CKM framework. These findings aim to inform targeted prevention strategies for mitigating the burden of severe cardiovascular events, diabetes-related complications, and kidney failure in this underserved population.

## 2 Materials and methods

### 2.1 Data source

This study is a cross-sectional analysis based on data from the China Cardiovascular Disease and Risk Factor Surveillance project, a nationwide survey (8) conducted from July 2020 to February 2021 using a stratified multistage sampling design. The present analysis focused on participants from the Guangxi Zhuang Autonomous Region.

### 2.2 Study population and sampling method

The original survey was approved by the relevant national and institutional ethics committees, and written informed consent was obtained from all participants at the time of data collection. The present secondary analysis of the Guangxi subset was approved by the Ethics Committee of the First Affiliated Hospital of Guangxi Medical University (Approval No. 2024-E706-01). All data used in this analysis were fully de-identified.

The study targeted adult residents aged≥18 years from Guangxi Zhuang Autonomous Region. A stratified multistage random sampling method was employed to ensure the representativeness of the study population. Initially, nine counties or urban districts in Guangxi were randomly selected as primary sampling units. From each selected county/urban area, two townships were randomly chosen as secondary sampling units. Within each township, three village committees were further randomly selected as tertiary sampling units (TSUs). Individuals aged ≥18 years within each TSU were stratified by age (per decade) and sex, and a proportionate number of participants were randomly selected from each stratum. Based on simple random sampling principles, the sample size was determined by considering an assumed prevalence, margin of error, design effect, and anticipated non-response rate according to the national surveillance protocol, resulting in an initial target sample of 9,600 residents. Participant recruitment and data collection were conducted at local community health service centers or temporary survey sites established in village committees or township clinics, following a unified national protocol.

### 2.3 Questionnaire survey, physical examination, and laboratory tests

Data collection was carried out by trained investigators following standardized protocols established by the National Center for Cardiovascular Diseases for the “China Cardiovascular Disease and Risk Factors Surveillance” project. A structured questionnaire was administered through face-to-face interviews to collect information on demographic characteristics, lifestyle factors, and medical history. Dietary intake was evaluated utilizing a one-month dietary recall method, with classification criteria aligned with the *Dietary Guidelines for Chinese Residents (2022)* to categorize food groups and nutrient intake (9).

All participants underwent a standardized physical examination, including measurements of height, weight, waist circumference (WC), and blood pressure. Blood pressure was measured using validated electronic sphygmomanometers (Omron HBP-1300, Omron Healthcare, Kyoto, Japan) with appropriate cuff sizes. Following a standardized protocol, three consecutive readings were taken after the participant had rested for at least 5 minutes, with the average value used for analysis. Body mass index (BMI) was calculated by dividing weight in kilograms by the square of height in meters (kg/m²). All devices were calibrated according to manufacturer instructions and project quality control guidelines.

Fasting blood samples were collected from all participants to measure levels of fasting blood glucose (FBG), glycated hemoglobin (HbA1c), total cholesterol (TC), high-density lipoprotein cholesterol (HDL-C), low-density lipoprotein cholesterol (LDL-C), triglycerides (TG), creatinine, and uric acid. The estimated glomerular filtration rate (eGFR) was calculated using the CKD-EPI equation (10). Morning urine samples were collected to assess creatinine and microalbumin levels and to calculate the urine albumin-to-creatinine ratio (UACR). Blood and urine samples were processed and transported under cold-chain conditions (2–8 °C) to Guangzhou KingMed Diagnostics for centralized analysis. Laboratory measurements were performed using standardized enzymatic or immunoturbidimetric methods.

The survey was conducted during the COVID-19 pandemic; however, standardized field procedures and quality control measures were implemented nationwide, and there was no evidence to suggest a substantial impact on data quality or measurement consistency.

### 2.4 Diagnostic criteria

CKM staging was defined according to the diagnostic criteria established by the American Heart Association (AHA) (11, 12), as detailed in **Supplementary Table 1**. Briefly, stage 0 represents individuals without CKM risk conditions; stage 1 includes individuals with excess or dysfunctional adiposity and/or prediabetes; stage 2 includes individuals with metabolic risk factors and/or CKD; stage 3 refers to subclinical cardiovascular disease or high predicted cardiovascular risk; and stage 4 indicates established clinical cardiovascular disease. CVD was categorized into hypertension (HTN) and non-HTN CVD. The latter comprised coronary artery disease (CAD), valvular heart disease, chronic heart failure, atrial fibrillation, cardiomyopathy, stroke (excluding lacunar infarction), and peripheral artery disease. These conditions were primarily identified based on self-reported medical history and verified through available medical records from local healthcare institutions where possible.

CKD was defined as either impaired kidney function (eGFR<60 mL/min/1.73 m²) or the presence of albuminuria (UACR≥30 mg/g) (13). Diabetes was defined as a self-reported history of diabetes, FBG level≥126 mg/dL (6.99 mmol/L), or HbA1c≥6.5% (14). Prediabetes was defined as FBG level of 100–125 mg/dL (5.6–6.9 mmol/L) or HbA1c of 5.7%–6.4% (14). Newly diagnosed diabetes referred to individuals who met diagnostic criteria during the study but had no prior diagnosis.

HTN was defined as systolic blood pressure (SBP)≥140 mmHg, diastolic blood pressure (DBP)≥90 mmHg, or a self-reported history of HTN (15). Newly diagnosed HTN was identified when blood pressure met or exceeded the diagnostic threshold during examination without a prior diagnosis. Previously diagnosed HTN was defined as a self-reported diagnosis or the use of antihypertensive medication within the past two weeks.

Dyslipidemia was defined as TC level≥240 mg/dL (6.22 mmol/L), HDL-C level ≤ 40 mg/dL (1.04 mmol/L), LDL-C level≥160 mg/dL (4.14 mmol/L), TG level≥200 mg/dL (2.26 mmol/L), or a history of hyperlipidemia (16).

Overweight was defined as BMI between 24.0 and 28.0 kg/m², and obesity was defined as BMI≥28 kg/m². Abdominal obesity was defined as WC≥85 cm for women and≥90 cm for men (17). The diagnostic criteria for metabolic syndrome were adapted from the harmonized international criteria (18), with modifications to reflect the specific anthropometric and metabolic characteristics of the Chinese population.

### 2.5 Statistical analysis

All statistical analyses were performed using R software, version 4.4.2 (R Foundation for Statistical Computing). The primary outcome was CKM stage (0–4), and its prevalence and distribution were described. Age- and sex-standardization was applied for overall prevalence estimates, while subgroup analyses were based on crude rates. Categorical variables are summarized as frequencies and were compared using chi-square tests or Fisher’s exact test. Univariate and multivariate logistic regression models were used to explore factors associated with moderate-to-advanced CKM (stage 2–4 vs. stage 0–1). A supplementary analysis for overall CKM (stage 1–4 vs. stage 0) was also conducted. Variables with P < 0.05 in univariate analyses were included in the multivariable models. Analyses were conducted using complete-case data, as the proportion of missing values for all variables was less than 5%. The complex survey design was not explicitly incorporated into the regression analyses. Subgroup analyses were performed according to sex, age group, residence, and ethnicity. Interaction effects were assessed by including interaction terms in the regression models. Forest plots for subgroup analyses were generated using Sangerbox 2.0, a comprehensive online data analysis platform (19).

To explore the interrelationships among CKM-related multimorbidities beyond traditional regression analyses, a network analysis was conducted. The network structure was estimated from binary disease variables (presence/absence) using the IsingFit method, as described by van Borkulo et al. (20). Diseases were selected based on the CKM framework and grouped into cardiovascular, metabolic, and kidney domains. To evaluate network edge accuracy and centrality stability, nonparametric bootstrapping with 1000 iterations was performed using the bootnet package, version 1.6 (Epskamp, 2018) (21). Centrality measures (including expected influence, betweenness, and closeness) were calculated to identify core nodes in the multimorbidity network. Major explanatory variables were categorized based on established clinical definitions or guideline-recommended cutoffs, as described above. A two-tailed *P* value < 0.05 was considered statistically significant.

## 3 Results

### 3.1 General characteristics of the study population

**Figure 1:**
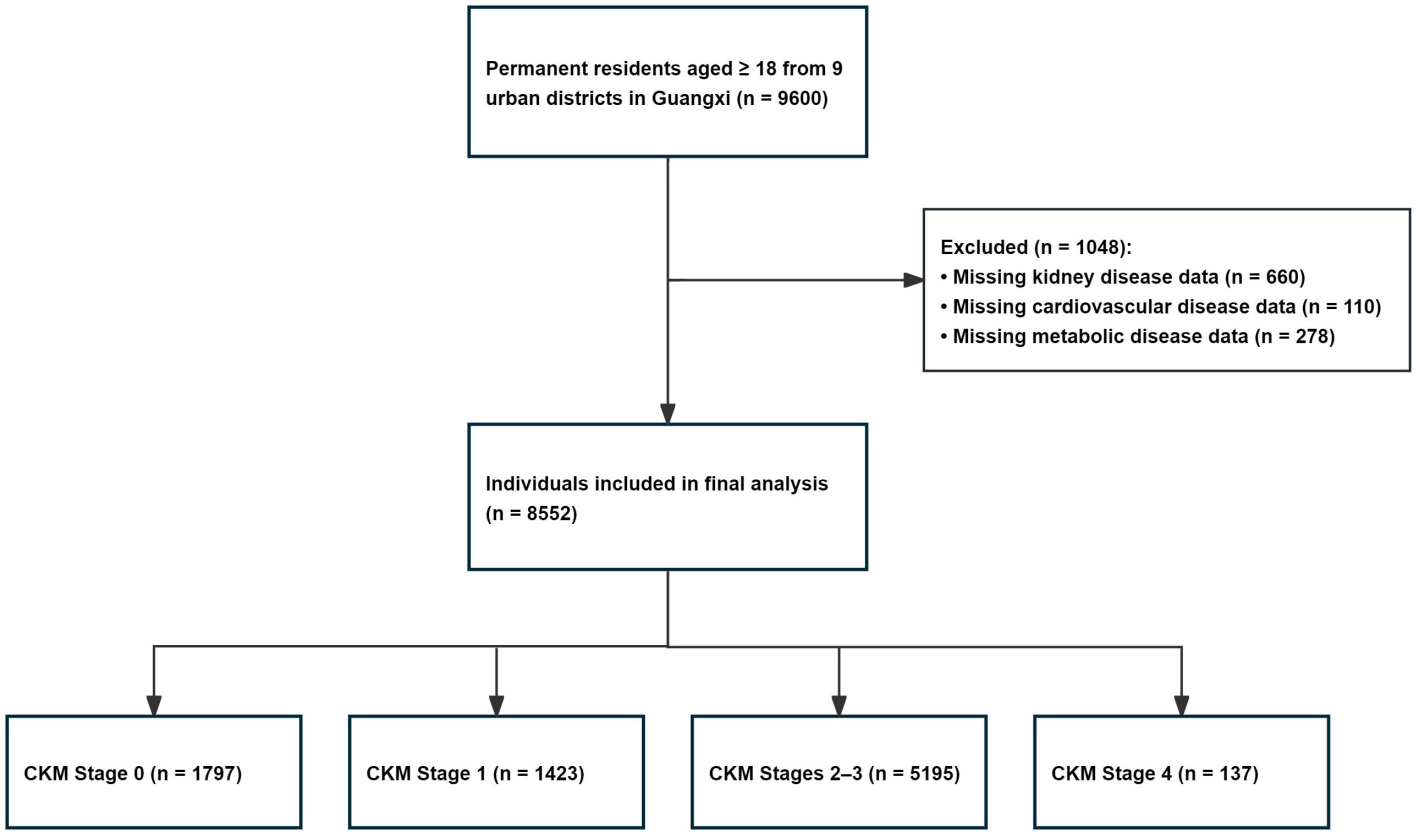
Participant screening flow diagram of this study. Abbreviations: CKM, cardiovascular-kidney-metabolic.

A total of 9,600 individuals were initially sampled, of whom 8,552 were included in the final analysis after excluding participants with missing data. The flowchart of participant screening is shown in **Figure 1**. Among the study population, 3,587 (41.9%) were male, and 4,965 (58.1%) were female. The participants’ age ranged from 18 to 99 years, with a median age of 50 years (IQR: 36–63). In terms of residence, 5,696 individuals (66.6%) were urban residents, and 2,856 (33.4%) resided in rural regions. With respect to ethnicity, 3,614 (42.3%) participants were Zhuang, and 4,938 (57.7%) belonged to other ethnic groups, including 4,706 individuals of Han ethnicity and 232 individuals from other minority groups such as Jing, Maonan, Mulao, and Shui. **Supplementary Table 2** summarizes the characteristics of the study population. The prevalence of chronic diseases was as follows: other CVD, 1.6% (137/8,552); CKD, 13.8% (1,176/8,552); obesity, 12.2% (1,043/8,552); abdominal obesity, 27.2% (2,328/8,552); HTN, 39.7% (3,394/8,552); diabetes, 12.1% (1,036/8,552); and metabolic syndrome, 23.0% (1,965/8,552).

### 3.2 Prevalence of CKM stages

Crude rate estimation showed that among 8,552 participants, 6,755 individuals (79.0%) were in CKM syndrome stages 1–4, and 1,797 (21.0%) were in stage 0. The specific distribution of crude prevalence by stage was as follows:1,423 (16.6%) were at stage 1, 5,195 (60.7%) at stages 2–3, and 137 (1.6%) at stage 4. After age- and sex-standardization using data from the 2020 Seventh National Population Census of China, the overall standardized prevalence of CKM stages 1–4 was 76.4% (95% CI: 75.5%–77.3%). The standardized prevalence by stage was as follows: stage 1(17.2%, 95% CI: 16.4%–18.1%), stages 2–3(57.7%, 95% CI: 56.7%–58.8%), and stage 4 (1.4%, 95% CI: 1.2%–1.7%). The characteristics of the study population across CKM stages are detailed in **Table 1**. **Table 2** presents the prevalence of CKM stages stratified by sex, residence, age, and ethnicity. Urban areas presented a higher prevalence of stages 0–1, whereas rural areas presented a higher prevalence of stages 2–4 (*P* < 0.05). Similarly, younger groups presented a higher prevalence of stages 0–1, whereas the older groups presented a higher prevalence of stages 2–4 (*P* < 0.05; see **Supplementary Figure 1**). The Zhuang population presented a greater prevalence of stage 1, whereas the other ethnic population presented a higher prevalence of stages 2–3 (*P* < 0.05). However, there was no statistically significant difference in the combined prevalence of stages 2–4 between the ethnic groups. Detailed age- and sex-specific distributions of CKM stages are shown in **Supplementary Figure 2**. Females had a higher prevalence of CKM 0–1 stage, while males had a higher prevalence of CKM 2–4 stage (*P* < 0.05).

**Table 1:** Characteristics of the study population across different CKM stages.

Characteristic	Total n (%) (n = 8552)	CKM				
		n (%)				
		Stage 0	Stage 1	Stages 2-3 (n = 5195 )	Stage 4	p value
		(n = 1797)	(n = 1423)		(n = 137)	
Sex						
Male	3587 (41.9)	570 (31.7)	511 (35.9)	2423 (46.6)	83 (60.6)	<0.001
Female	4965 (58.1)	1227 (68.3)	912 (64.1)	2772 (53.4)	54 (39.4)	
Age (y)						
18-<45	3371 (39.4)	1333 (74.2)	692 (48.6)	1335 (25.7)	11 (8.0)	<0.001
45-64	3207 (37.5)	353 (19.6)	591 (41.5)	2200 (42.4)	63 (46.0)	
≥65	1974 (23.1)	111 (6.2)	140 (9.8)	1660 (32.0)	63 (46.0)	
Residence						
Urban	5696 (66.6)	1327 (73.9)	1000 (70.3)	3295 (63.4)	74 (54.0)	<0.001
Rural	2856 (33.4)	470 (26.2)	423 (29.7)	1900 (36.6)	63 (46.0)	
Ethnicity						
Zhuang	3614 (42.3)	763 (42.5)	647 (45.5)	2150 (41.4)	54 (39.4)	0.044
Non-Zhuang	4938 (57.7)	1034 (57.5)	776 (54.5)	3045 (58.6)	83 (60.6)	
Educational Level						
No or primary education	3072 (35.9)	279 (15.5)	393 (27.6)	2334 (44.9)	66 (48.2)	<0.001
Middle school	3899 (45.6)	908 (50.5)	724 (50.9)	2205 (42.4)	62 (45.3)	
College/University	1581 (18.5)	610 (34.0)	306 (21.5)	656 (12.6)	9 (6.6)	
Per capita income						
(last year, 10,000 CNY)						
<2	2226 (26.0)	340 (18.9)	352 (24.7)	1489 (28.7)	45 (32.9)	<0.001
2-<3	1959 (22.9)	391 (21.8)	295 (20.7)	1234 (23.8)	39 (28.5)	
3-<5	2178 (25.5)	485 (27.0)	382 (26.8)	1282 (24.7)	29 (21.2)	
≥5	2189 (25.6)	581 (32.3)	394 (27.7)	1190 (22.9)	24 (17.5)	
Smoking status						
Yes	1573 (18.4)	240 (13.4)	221 (15.5)	1080 (20.8)	32 (23.4)	<0.001
No	6964 (81.4)	1551 (86.3)	1200 (84.3)	4108 (79.2)	105 (76.6)	
Drinking consumption						
Yes	1729 (20.2)	263 (14.6)	253 (17.8)	1177 (22.7)	36 (26.3)	<0.001
No	6808 (79.6)	1528 (85.0)	1168 (82.1)	4011 (77.3)	101 (73.7)	
Vegetable intake (<300 g/d)						
Yes	5347 (62.5)	1210 (67.3)	911 (64.0)	3139 (60.5)	87 (63.5)	<0.001
No	3196 (37.4)	584 (32.5)	510 (35.8)	2052 (39.5)	50 (36.5)	
Fruit intake (<200 g/d)						
Yes	6024 (70.4)	1228 (68.3)	970 (68.2)	3722 (71.7)	104 (75.9)	0.006
No	2519 (29.5)	566 (31.5)	451 (31.7)	1469 (28.3)	33 (24.1)	
Red meat intake (≥200 g/d)						
Yes	3240 (37.9)	608 (33.8)	523 (36.8)	2065 (39.8)	44 (32.1)	<0.001
No	5303 (62.0)	1186 (66.0)	898 (63.1)	3126 (60.2)	93 (67.9)	
Physical inactivity						
Yes	1936 (22.6)	441 (24.5)	263 (18.5)	1189 (23.1)	43 (31.4)	<0.001
No	6553 (76.6)	1343 (74.7)	1149 (80.7)	3967 (76.9)	94 (68.6)	
BMI (kg/m²)						
<18.5	509 (6.0)	252 (14.0)	64 (4.5)	190 (3.7)	3 (2.2)	<0.001
18.5- <24	4156 (48.6)	1468 (81.7)	493 (34.7)	2139 (41.2)	56 (40.9)	
24- <28	2844 (33.3)	56 (3.1)	707 (49.7)	2028 (39.0)	53 (38.7)	
≥28	1043 (12.2)	21 (1.2)	159 (11.2)	838 (16.1)	25 (18.3)	
Abdominal obesity						
Yes	2328 (27.2)	0 (0.0)	389 (27.3)	1881 (36.2)	58 (42.3)	<0.001
No	6224 (72.8)	1797 (100.0)	1034 (72.7)	3314 (63.8)	79 (57.7)	
HTN						
No HTN	5158 (60.3)	1797 (100.0)	1423 (100.0)	1907 (36.7)	31 (22.6)	<0.001
Newly diagnosed	2100 (24.6)	0 (0.0)	0 (0.0)	2068 (39.8)	32 (23.4)	
Previously diagnosed	1294 (15.1)	0 (0.0)	0 (0.0)	1220 (23.5)	74 (54.0)	
Diabetes						
No diabetes	4583 (53.6)	1797 (100.0)	660 (46.4)	2088 (40.2)	38 (27.7)	<0.001
Prediabetes	2933 (34.3)	0 (0.0)	763 (53.6)	2104 (40.5)	66 (48.2)	
Newly diagnosed	743 (8.7)	0 (0.0)	0 (0.0)	729 (14.0)	14 (10.2)	
Previously diagnosed	293 (3.4)	0 (0.0)	0 (0.0)	274 (5.3)	19 (13.9)	
Dyslipidemia						
Yes	2817 (32.9)	124 (6.9)	217 (15.2)	2416 (46.5)	60 (43.8)	<0.001
No	5735 (67.1)	1673 (93.1)	1206 (84.8)	2779 (53.5)	77 (56.2)	
Hyperuricemia						
Yes	1993 (23.3)	205 (11.4)	237 (16.7)	1497 (28.8)	54 (39.4)	<0.001
No	6559 (76.7)	1592 (88.6)	1186 (83.3)	3698 (71.2)	83 (60.6)	
History of other CVD						
Yes	137 (1.6)	0 (0.0)	0 (0.0)	0 (0.0)	137 (100.0)	<0.001
No	8415 (98.4)	1797 (100.0)	1423 (100.0)	5195 (100.0)	0 (0.0)	
UACR ≥ 30 mg/g						
Yes	864 (10.1)	12 (0.7)	7 (0.5)	820 (15.8)	25 (18.3)	<0.001
No	7688 (89.9)	1785 (99.3)	1416 (99.5)	4375 (84.2)	112 (81.8)	
eGFR <60 ml/min/1.73m^2^						
Yes	443 (5.2)	0 (0.0)	0 (0.0)	418 (8.1)	25 (18.3)	<0.001
No	8109 (94.8)	1797 (100.0)	1423 (100.0)	4777 (92.0)	112 (81.8)	

BMI, Body Mass Index; HTN, hypertension; UACR, urine albumin-to-creatinine ratio; eGFR, Estimated Glomerular Filtration Rate; CKM, Cardiovascular-Kidney-Metabolic. CVDs: Cardiovascular Diseases..

^a^ Data with cell counts < 5 were analyzed using Fisher’s exact test; data with cell counts ≥ 5 were analyzed using the Chi-square test.

**Table 2:** Prevalence of CKM stages by sex, residence, age, and ethnicity.

CKM Stages	Sex,				Residence,				Age (y),					Ethnicity,				Total n (%) (n=8552)
	n (%)				n (%)				n (%)					n (%)				
	Female	Male	χ²	p value	Urban (n=5696)	Rural (n=2856)	χ²	p value	18 to 44 (n = 3371)	45 to 64 (n = 3207)	≥65 (n = 1974)	χ²	p value	Zhuang (n=3614)	Non-Zhuang (n=4938)	χ²	p value	
Stage 0	1227 (24.7)	570 (15.9)	97.66	<0.001	1327 (23.3)	470 (16.5)	53.63	<0.001	1333 (39.5)	353 (11.0)	111 (5.6)	1172.54	<0.001	763 (21.1)	1034 (20.9)	0.04	0.846	1797 (21.0)
Stage 1	912 (18.4)	511 (14.3)	25.52	<0.001	1000 (17.6)	423 (14.8)	10.34	0.001	692 (20.5)	591 (18.4)	140 (7.1)	173.87	<0.001	647 (17.9)	776 (15.7)	7.20	0.007	1423 (16.6)
Overweight/Obesity	570 (62.5)	296 (57.9)	2.88	0.090	606 (60.6)	260 (61.5)	0.09	0.760	469 (67.8)	346 (58.5)	51 (36.4)	50.30	<0.001	386 (59.7)	480 (61.9)	0.71	0.398	866 (60.9)
Abdominal Obesity	250 (27.4)	139 (27.2)	0.01	0.932	278 (27.8)	111 (26.2)	0.36	0.546	191 (27.6)	171 (28.9)	27 (19.3)	5.35	0.069	181 (28.0)	208 (26.8)	0.24	0.622	389 (27.3)
Prediabetes	472 (51.8)	291 (57.0)	3.55	0.060	534 (53.4)	229 (54.1)	0.06	0.799	272 (39.3)	371 (62.8)	120 (85.7)	134.91	<0.001	362 (56.0)	401 (51.7)	2.59	0.107	763 (53.6)
Stages 2-3	2772 (55.8)	2423 (67.6)	119.93	<0.001	3295 (57.9)	1900 (66.5)	60.09	<0.001	1335 (39.6)	2200 (68.6)	1660 (84.1)	1166.20	<0.001	2150 (59.5)	3045 (61.7)	4.14	0.042	5195 (60.7)
Hypertriglyceridemia	1405 (50.7)	1540 (63.6)	87.25	<0.001	1895 (57.5)	1050 (55.3)	2.48	0.115	962 (72.1)	1293 (58.8)	690 (41.6)	286.98	<0.001	1223 (56.9)	1722 (56.6)	0.06	0.812	2945 (56.7)
HTN	1841 (66.4)	1447 (59.7)	24.94	<0.001	1980 (60.1)	1308 (68.8)	39.72	<0.001	483 (36.2)	1438 (65.4)	1367 (82.4)	685.93	<0.001	1301 (60.5)	1987 (65.3)	12.20	<0.001	3288 (63.3)
Metabolic Syndrome	1052 (38.0)	859 (35.5)	3.47	0.062	1185 (36.0)	726 (38.2)	2.62	0.106	402 (30.1)	877 (39.9)	632 (38.1)	35.71	<0.001	804 (37.4)	1107 (36.4)	0.59	0.444	1911 (36.8)
Diabetes	514 (18.5)	489 (20.2)	2.23	0.135	604 (18.3)	399 (21.0)	5.51	0.019	483 (36.2)	1438 (65.4)	1367 (82.4)	111.34	<0.001	452 (21.0)	551 (18.1)	6.93	0.008	1003 (19.3)
CKD	652 (23.5)	483 (19.9)	9.74	0.002	731 (22.2)	404 (21.3)	0.60	0.439	166 (12.4)	393 (17.9)	576 (34.7)	250.29	<0.001	446 (20.7)	689 (22.6)	2.62	0.106	1135 (21.9)
Stage 4	54 (1.1)	83 (2.3)	19.87	<0.001	74 (1.30)	63 (2.2)	9.92	0.002	11 (0.3)	63 (2.0)	63 (3.2)	69.11	<0.001	54 (1.5)	83 (1.7)	0.46	0.497	137 (1.6)
CAD	19 (35.2)	35 (42.2)	0.67	0.414	36 (48.7)	18 (28.6)	5.74	0.017	4 (36.4)	18 (28.6)	32 (50.8)	6.54^a^	0.033	19 (35.2)	35 (42.2)	0.67	0.414	54 (39.4)
Heart Disease	30 (55.6)	53 (63.9)	0.94	0.331	51 (68.9)	32 (50.8)	4.68	0.030	10 (90.9)	35 (55.6)	38 (60.3)	4.99^a^	0.083	32 (59.3)	51 (61.5)	0.07	0.798	83 (60.6)
Stroke	25 (46.3)	32 (38.6)	0.81	0.369	25 (33.8)	32 (50.8)	4.05	0.044	1 (9.1)	28 (44.4)	28 (44.4)	5.36^a^	0.074	23 (42.6)	34 (41.0)	0.04	0.850	57 (41.6)

Hypertriglyceridemia is defined as triglycerides ≥1.52 mmol/L (134.51 mg/dL). The SI conversion factor for triglycerides is 1 mg/dL = 0.0113 mmol/L. Coronary artery disease (CAD) was defined based on a self-reported history of myocardial infarction, hospitalization for unstable angina, percutaneous coronary intervention (PCI), or coronary artery bypass grafting (CABG). Heart disease included self-reported history of valvular heart disease, chronic heart failure, atrial fibrillation, cardiomyopathy, and coronary artery disease. Stroke was defined based on a self-reported history of subarachnoid hemorrhage, cerebral hemorrhage, cerebral infarction, or other cerebrovascular events, excluding lacunar infarction. Abbreviations: HTN, hypertension; CKD, chronic kidney disease; CAD, coronary artery disease. ^a^ Data with cell counts < 5 were analyzed using Fisher’s exact test; data with cell counts ≥ 5 were analyzed using the Chi-square test.

Analysis of awareness and control rates of complications showed the following: among HTN patients, the awareness rate was 37.8% (1,294/3,394), the treatment rate of 31.3% (1,063/3,394) and control rate was 8.8% (298/3,394); among diabetes patients, the awareness rate was 28.3% (293/1,036), and the glycemic control rate (HbA1c<7%) was 61.2% (634/1,036); among dyslipidemia patients, the awareness rate was 7.0% (196/2,817), with a control rate of 3.5% (98/2,817); the awareness rate of CKD was only 1.4% (17/1,176).

### 3.3 Associated factors for moderate-to-advanced CKM (stages 2-4)

Results of the univariate and multivariate regression analyses of factors associated with CKM stages 2–4 are shown in **Table 3**. Multivariate analysis indicated that factors independently associated with moderate-to-advanced CKM included male sex (OR = 1.75, 95% CI: 1.55–1.97; *P* < 0.001), age (45–64 years: OR = 3.04, 95% CI: 2.72–3.41; ≥65 years: OR = 7.52, 95% CI: 6.38–8.89; both P < 0.001), rural residence (OR = 1.48, 95% CI: 1.32–1.65; *P* < 0.001), education level (middle school: OR = 1.25, 95% CI: 1.09–1.43; primary education or below: OR = 2.18, 95% CI: 1.87–2.55; both *P* < 0.001), alcohol consumption (OR = 1.30, 95% CI: 1.12–1.50; *P* < 0.001), fruit intake <200 g/d (OR = 1.15, 95% CI: 1.01–1.30; *P* = 0.031), red meat intake ≥200 g/d (OR = 1.15, 95% CI: 1.02–1.30; *P* = 0.021), and hyperuricemia (OR = 2.54, 95% CI: 2.24–2.90; *P* < 0.001). Results of univariate and multivariate regression analyses of factors associated with CKM stages 1–4 are shown in **Supplementary Table 3**, and the composition of associated factors was broadly consistent with that observed for CKM stages 2–4 (**Table 3**).

**Table 3:** Multivariate binary logistic regression analysis of factors associated with CKM syndrome stages 2-4.

Characteristic	Univariate analysis		Multivariate analysis	
	*OR (95%CI)*	P value	*OR (95%CI)*	P value
Sex				
Female	1.00 (Reference)		1.00 (Reference)	
Male	1.75 (1.60 - 1.92)	<0.001	1.75 (1.55 - 1.97)	<0.001
Age, y				
18-44	1.00 (Reference)		1.00 (Reference)	
45-64	3.61 (3.26 - 4.00)	<0.001	3.04 (2.72 - 3.41)	<0.001
≥65	10.33 (8.90 - 11.99)	<0.001	7.52 (6.38 - 8.89)	<0.001
Ethnicity				
Zhuang	1.00 (Reference)			
other	1.11 (1.01 - 1.21)	0.026		
Educational Level				
College/University	1.00 (Reference)		1.00 (Reference)	
Secondary school	1.91 (1.70 - 2.15)	<0.001	1.25 (1.09 - 1.43)	<0.001
Primary education or below	4.92 (4.31 ~ 5.61)	<0.001	2.18 (1.87 - 2.55)	<0.001
Residence				
Urban	1.00 (Reference)		1.00 (Reference)	
Rural	1.52 (1.38 - 1.67)	<0.001	1.48 (1.32 - 1.65)	<0.001
income (last year, 10,000 CNY)				
2-<3	1.00 (Reference)			
<2	1.19 (1.05 - 1.36)	0.007		
3-<5	0.81 (0.72 - 0.92)	0.001		
≥5	0.67 (0.59 - 0.76)	<0.001		
Smoking status				
No	1.00 (Reference)			
Yes	1.58 (1.40 - 1.77)	<0.001		
Alcohol consumption				
No	1.00 (Reference)		1.00 (Reference)	
Yes	1.54 (1.38- 1.73)	<0.001	1.30 (1.12 - 1.50)	<0.001
Vegetable intake <300 g/d				
No	1.00 (Reference)			
Yes	0.79 (0.72 - 0.87)	<0.001	0.80 (0.72 - 0.89)	<0.001
Fruit intake <200 g/d				
No	1.00 (Reference)			
Yes	1.18 (1.07 - 1.30)	<0.001	1.15 (1.01 - 1.30)	0.031
Red meat intake ≥200 g/d				
No	1.00 (Reference)		1.00 Reference	
Yes	1.21 (1.10 - 1.32)	<0.001	1.15 (1.02 - 1.30)	0.021
Physical inactivity				
No	1.00 (Reference)			
Yes	1.07 (0.97 - 1.19)	0.184		
Hyperuricemia				
No	1.00 (Reference)		1.00 (Reference)	
Yes	2.58 (2.29 - 2.90)	<0.001	2.54 (2.24 - 2.90)	<0.001

CKM, Cardiovascular-Kidney-Metabolic.

### 3.4 Subgroup analyses

**Figure 2:**
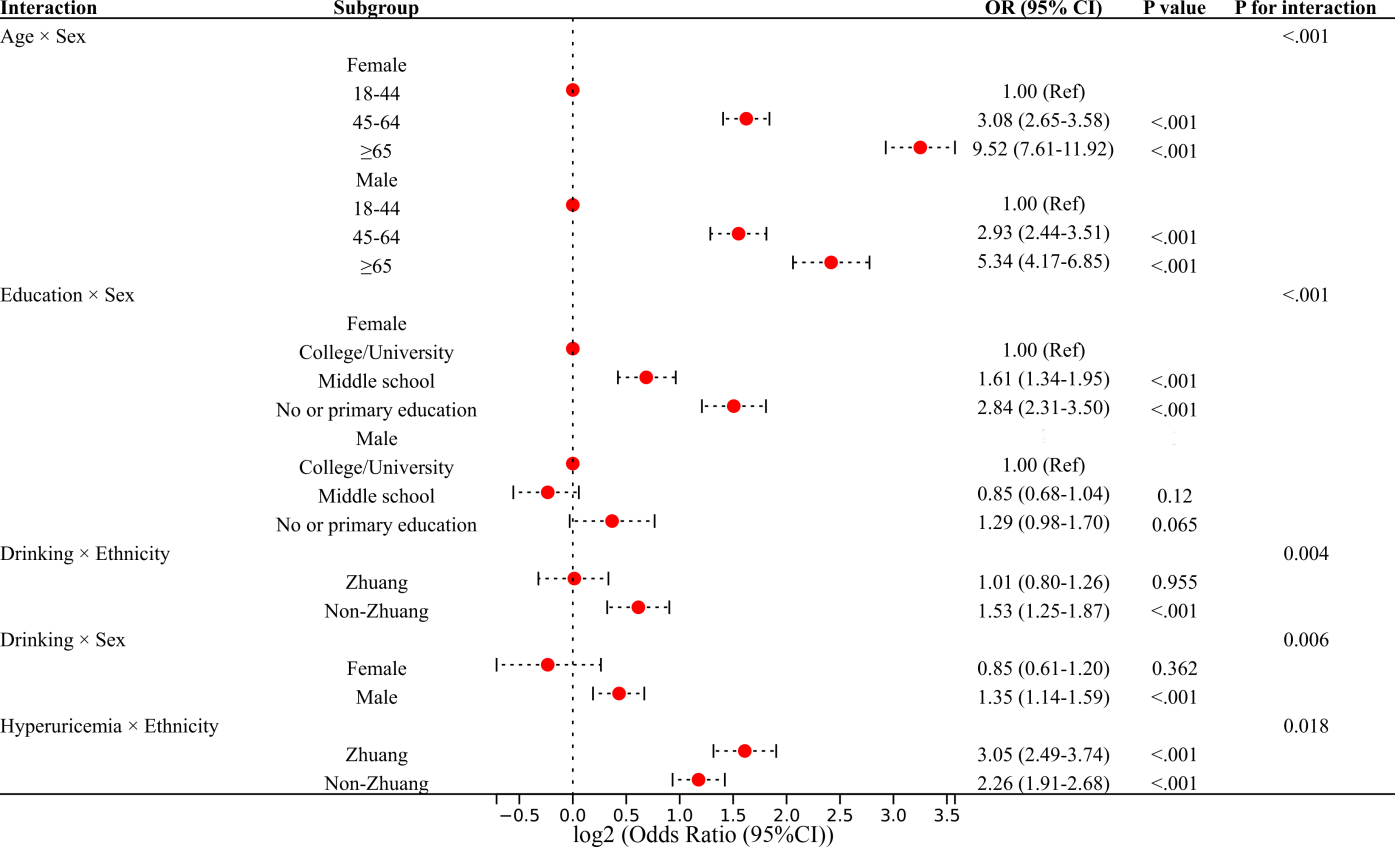
Stratified analysis of factors associated with CKM syndrome stages 2–4 by subgroups ( p for interaction < 0.05 ). Odds ratios (ORs) and 95% confidence intervals (CIs) are plotted on a log_2_ scale. Each model included one interaction term between the stratification variable and the exposure of interest. All models were adjusted for age group, sex, ethnicity, education level, urban or rural residence, income level, smoking status, fruit intake, vegetable intake, red meat intake, hyperuricemia, and drinking status. Abbreviations: OR, odds ratio; CI, confidence interval; CKM, cardiovascular–kidney–metabolic.

Subgroup analyses revealed generally consistent interaction patterns between CKM stages 2–4 and stages 1–4. Significant interactions between education level and sex were observed in both models (*P* for interaction <0.001), with a stronger association between lower education and CKM in females than in males. Similarly, the interaction between hyperuricemia and ethnicity was significant in both analyses (*P* for interaction = 0.018 for stages 2–4 and 0.023 for stages 1–4), showing a stronger effect among Zhuang participants. Notably, certain interactions differed by CKM stage: the interactions between alcohol consumption and both ethnicity and sex were significant in stages 2–4 model, whereas interaction between hyperuricemia and age was significant only in CKM stages 1–4 model. No significant interaction effects were observed for residence. These findings suggest stage-specific variations in CKM association patterns. Forest plots of interaction analyses for CKM stages 2–4 and stages 1–4 are shown in **Figure 2** and **Supplementary Figure 3**, respectively.

### 3.5 CKM multimorbidity network

**Figure 3:**
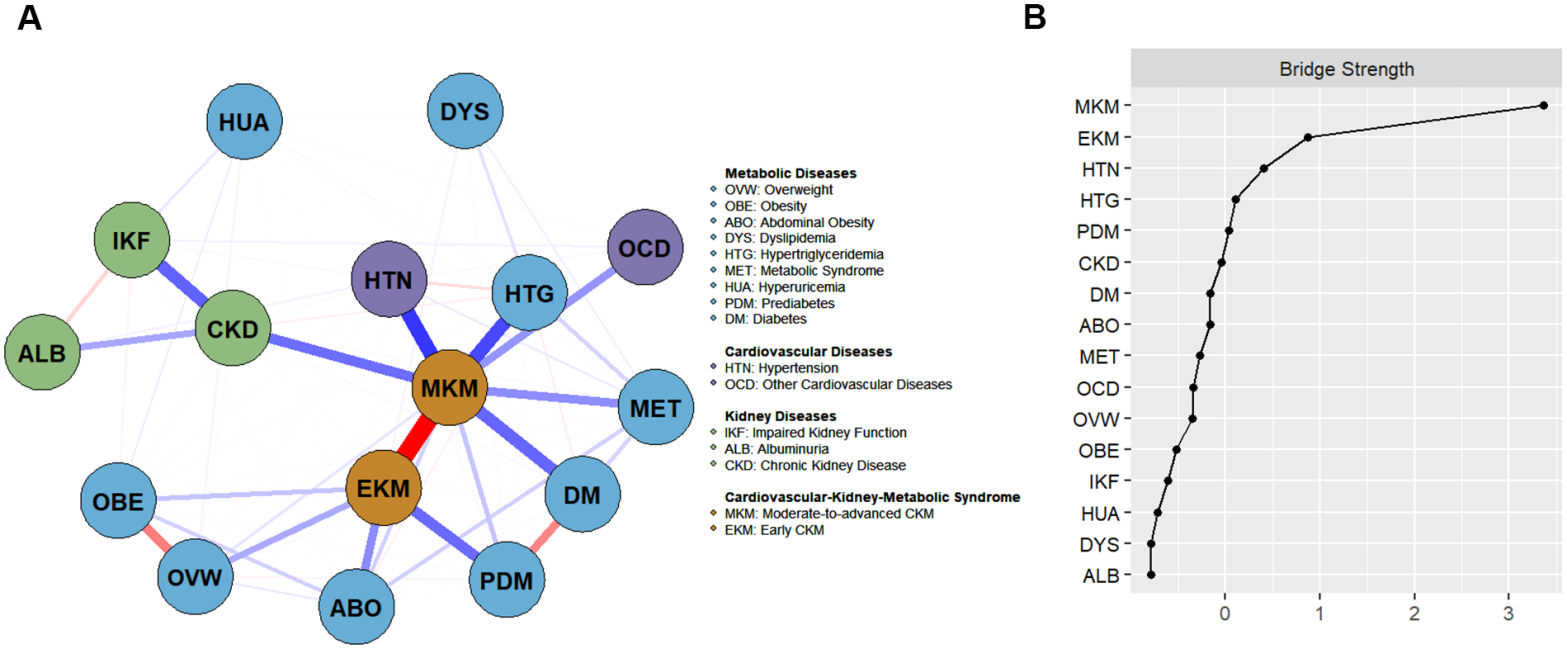
**(A)** Multimorbidity network analysis diagram of CKM syndrome; **(B)** Bridge strength centrality in the CKM network structure. The network diagram illustrates the relationships between CKM (Cardiovascular-Kidney-Metabolic Syndrome) and related diseases, including metabolic, cardiovascular, and kidney conditions. It was estimated using IsingFit with regularization to reduce spurious connections. Blue edges indicate positive conditional dependencies, while red edges indicate negative dependencies. Edge thickness reflects regression coefficient strength. Abbreviations: CKM, Cardiovascular-Kidney-Metabolic; EKM, Early Cardiovascular-Kidney-Metabolic; MKM, Moderate-to-advanced Cardiovascular-Kidney-Metabolic.

In this study, CVD was categorized as HTN and non-HTN CVD, a classification distinct from the AHA CKM framework (which classifies HTN separately). This approach ensured analytical stability in the network analysis by reducing sparsity due to the relatively low prevalence of individual non-HTN CVD conditions. **Figure 3A** presents the CKM network analysis diagram, showing interrelationships among cardiovascular, renal, and metabolic disorders. Moderate-to-advanced CKM was identified as the most central node, demonstrating significant positive conditional dependencies with HTN (β = 11.723) and hypertriglyceridemia (β = 10.656) (**Supplementary Table 4**). **Figure 3B** shows that HTN serves as a critical bridging node, linking cardiovascular, renal, and metabolic diseases (bridge strength z score = 0.399). **Supplementary Figure 4** presents the expected influence centrality values of key nodes, with CKD, metabolic syndrome, abdominal obesity, and HTN displaying relatively high centrality (z-scores of 0.999, 0.717, 0.373, and 0.143, respectively). Results of bootstrap 95% CI for edge weights are shown in **Supplementary Figure 5**, which confirms the stability of edge estimates. The centrality measures demonstrated excellent stability (CS coefficient = 0.75), maintaining consistent node rankings after the exclusion of 75% of the sample (**Supplementary Figure 6**). Comparative analysis of urban and rural networks (**Supplementary Figure 7**) revealed significant differences in overall network strength (urban: 165.34, rural: 137.97, S = 27.38, *P* = 0.030). However, there were no significant structural differences observed between urban and rural networks or in pairwise disease connection strengths.

## 4 Discussion

Since the AHA formalized the CKM syndrome framework (12), increasing attention has been directed toward the syndemic interplay among CVD, CKD, and metabolic disorders, particularly with respect to their epidemiological trajectories and population burden. However, how these conditions cluster and interact across CKM stages in multi-ethnic, socioeconomically underdeveloped regions remains insufficiently characterized. In this context, our population-based study in Guangxi—an autonomous region in southern China characterized by distinctive karst geography, a subtropical climate, and a large Zhuang ethnic population (22)—provides comprehensive, stage-specific epidemiological evidence on the prevalence, distribution, and interrelationships of CKM syndrome in this understudied setting.

Our study revealed a distinct epidemiological profile of CKM syndrome in the multi-ethnic population of Guangxi. The overall adjusted prevalence of CKM (stages 1–4) was 76.4%, with the majority of affected individuals in stages 2–3 (57.7%), followed by stage 1 (17.2%) and stage 4 (1.4%). This overall prevalence is notably lower than the estimates reported in two major national cohorts: the Pinggu Study (90.1%) and the CHARLS study (82.6%) (23). Consistent with this pattern, the prevalences of key CKM component conditions in our population—including diabetes (12.1% vs. 16.4–16.6%) and non-hypertensive cardiovascular disease (1.6% vs. 8.9–13.4%)—were also lower than those in both reference cohorts. When compared to data from the U.S. NHANES, the distribution across CKM stages in Guangxi showed marked differences. The U.S. population had a lower proportion at stage 0 (10.6%) and a higher proportion at stages 2–4 (63.6%) (3). In contrast, our cohort showed a higher prevalence of stage 0 (23.6%) and a lower prevalence of stages 2–4 (59.1%). This relatively favorable profile in Guangxi may be linked to a more advantageous cardiometabolic risk factor distribution. Specifically, our population exhibited lower mean BMI (23.8 kg/m² vs. 26.1 kg/m² in the Pinggu cohort (23)), along with lower rates of obesity and diabetes—key drivers of CKM progression. Despite this, the age- and sex-related progression patterns were consistent with broader epidemiological trends. We observed a pronounced age-related increase in advanced-stage CKM, and male participants had significantly higher rates than females, aligning with findings from U.S. studies (24) and other Chinese cohorts (23). This underscores that while the baseline risk profile may vary, the fundamental drivers of disease progression remain potent.

We found distinct residence patterns in the distribution of cardiometabolic conditions. HTN, diabetes, dyslipidemia, and other CVD were more prevalent in rural areas, whereas hyperuricemia was more common among urban residents. These contrasting risk profiles likely contribute to the higher burden of CKM stages 2–4 observed in rural populations. Several contextual factors may underlie these disparities. Rural residents in Guangxi generally face lower health awareness and reduced access to preventive healthcare services, which may delay the detection and management of cardiometabolic conditions. Consistent with this interpretation, our study observed higher rates of red meat consumption, physical inactivity, overweight or obesity, and albuminuria among rural residents—factors that collectively reflect the challenges of chronic disease control during rapid urbanization and epidemiological transition. Notably, residence divergence in CKM stage 4 complications was observed: urban patients had higher coronary event rates, whereas rural patients had greater stroke prevalence. This necessitates region-specific strategies: enhanced HTN management and stroke prevention in rural areas versus optimized coronary disease secondary prevention in urban populations.

Previous studies have reported ethnic differences in CKM prevalence across stages (3, 24). Consistent with this literature, we observed that non-Zhuang participants exhibited a higher prevalence of CKM stages 2–3 compared with the Zhuang population. This difference may be partially explained by the lower prevalence of HTN among the Zhuang, potentially related to favorable traditional dietary patterns characterized by higher intake of rice and fresh leafy vegetables and lower consumption of dairy products and desserts (25). However, after adjusting for confounders, ethnicity showed no significant association with CKM, suggesting that behavioral and related factor variations, rather than inherent ethnic differences, underlie these disparities. These findings highlight the importance of prioritizing interventions targeting modifiable factors associated with CKM, rather than ethnicity per se, in multi-ethnic regions such as Guangxi.

Beyond individual- and group-level differences, broader regional socioeconomic and cultural contexts may further shape the distribution of factors associated with CKM in Guangxi. Despite maintaining traditional southern Chinese dietary patterns characterized by rice-based staples and high vegetable intake (26), Guangxi remains economically and educationally underdeveloped. The region’s per capita GDP in 2022 ranked near the bottom nationally, at approximately 61% of the national average (27, 28), and higher education enrollment rates also lag behind national levels (29, 30). In our study population, lower educational attainment and lower income were common, reflecting persistent structural constraints that may limit health literacy, access to preventive care, and adoption of healthy lifestyles. Collectively, these socioeconomic disadvantages provide an important contextual backdrop for the clustering of cardiometabolic risk factors and the relatively higher burden of moderate-to-advanced CKM observed in this region, underscoring the need for prevention strategies that are responsive to local population characteristics.

Our study identified several key modifiable factors associated with CKM in Guangxi’s population, including educational attainment, specific dietary components, alcohol consumption, and hyperuricemia. These factors not only demonstrate independent associations with CKM but also exhibit complex interactions with demographic characteristics, highlighting the need for targeted intervention strategies.

Lower educational attainment was significantly associated with CKM, consistent with previous evidence linking education to cardiometabolic health (31, 32). This association is likely mediated through multiple pathways, including inadequate health knowledge, limited access to health information, and adoption of suboptimal health behaviors. Interestingly, the protective effect of higher education was particularly pronounced among women in our stratified analyses: women with primary education had significantly higher odds of CKM (OR 3.21) compared to those with university education, whereas no significant educational gradient was observed among men (*P* for interaction < 0.001). This sex-specific pattern aligns with findings from other Chinese cohorts (32) and may reflect gender differences in health-seeking behaviors, social roles, and cumulative life-course exposures.

Regarding specific dietary components, our findings revealed complex associations with CKM. Although increased consumption of fruits and vegetables is widely recommended for cardiometabolic health (33), we observed an apparently counterintuitive association between lower vegetable intake (<300 g/d) and lower odds of CKM (OR = 0.8). Further analysis suggested this finding may be explained by confounding factors: individuals with lower vegetable intake also consumed less red meat (27.1% vs. 56.0% consuming ≥200 g/d) and were more likely to be younger (41.7% vs. 35.5% aged 18–44 years). These findings underscore the importance of considering overall dietary patterns and demographic characteristics when interpreting associations with CKM. Furthermore, the strong association between red meat consumption and moderate-to-advanced CKM progression emphasizes the need for dietary interventions promoting healthier protein sources, particularly in rural areas where red meat consumption is more prevalent.

Alcohol consumption demonstrated heterogeneous effects across demographic subgroups. Our findings revealed that the association between alcohol use and CKM was more evident among men and non-Zhuang individuals, whereas no significant association was observed among women or Zhuang participants, consistent with previous reports of sex- and ethnicity-specific associations between alcohol consumption and cardiovascular outcomes (34). This heterogeneity suggests that alcohol recommendations should consider population-specific characteristics rather than adopting universal guidelines.

Taken together, these findings underscore the central role of modifiable health behaviors in shaping CKM burden. The updated Life’s Essential 8 (LE8) framework proposed by the AHA provides a comprehensive approach to cardiovascular health, encompassing diet quality, physical activity, smoking, sleep, weight management, lipid levels, blood glucose, and blood pressure (33). Although LE8 was not specifically developed for CKM management, its core components closely align with the key behavioral and metabolic determinants of CKM identified in our study. Recent evidence suggests that greater adherence to LE8 is associated with reduced biological aging (35), improved cardiovascular health (36, 37), and lower all-cause mortality (36, 38). Our findings, therefore, lend further support to the relevance of integrated, behavior-centered prevention strategies for mitigating the burden of CKM in the general population.

Hyperuricemia emerged as a significant biological factor associated with CKM, with important variations across demographic subgroups. The hyperuricemia-CKM association was significantly stronger among Zhuang participants (OR 3.52) compared to non-Zhuang participants (OR 2.31; *P* for interaction = 0.023). This ethnic difference may reflect genetic variations in uric acid metabolism among southern Chinese minorities, as suggested by recent population studies (39). Additionally, the detrimental effect of hyperuricemia intensified with age: the association was substantially stronger among participants aged ≥65 years (OR 4.60) compared to those aged 18–44 years (OR 2.27; *P* for interaction = 0.009). This age-related intensification likely reflects multiple factors, including age-related declines in renal function that impair uric acid excretion (40), and increased comorbidity burden. Recent evidence supports causal links between elevated serum uric acid and cardiovascular, renal, and metabolic diseases in middle-aged and older adults (41).

Network analysis of multimorbidity in our study identified HTN as the central hub bridging cardiovascular, renal, and metabolic systems, suggesting that blood pressure control may help stabilize the CKM network. This central role of HTN is likely mediated by its bidirectional pathophysiological mechanisms: HTN activates the renin–angiotensin–aldosterone system (RAAS), leading to vascular injury and renal dysfunction, while simultaneously promoting insulin resistance and metabolic dysregulation (42, 43). Through these interconnected pathways, HTN serves as a critical node linking and potentiating dysfunction across all three systems. Compared with rural populations, urban populations demonstrated significantly greater overall disease connectivity, suggesting tighter clustering of cardiometabolic conditions. This pattern may imply distinct intervention priorities, with integrated multimorbidity management particularly relevant in urban settings, while targeted factor control may remain essential in rural areas.

Several potential sources of bias should be considered in this study. Selection bias was minimized through a stratified multistage random sampling design with efforts to account for non-response. Information bias was reduced by standardized face-to-face interviews conducted by trained investigators and verification of major cardiovascular conditions using medical records whenever available. Measurement bias was controlled through unified protocols, calibrated instruments, and repeated measurements. In addition, laboratory variability was minimized through centralized testing using standardized methods and strict quality control procedures. These measures collectively enhanced the reliability and validity of the study findings.

Despite these efforts, several limitations should be acknowledged. First, the cross-sectional design precludes causal inference. Second, some variables were obtained through self-reported questionnaires, which may introduce recall bias or reporting bias. Third, although multiple covariates were adjusted for, some potential confounders, such as detailed physical activity measures, medication use, and family history of cardiometabolic diseases, were not fully accounted for, which may have resulted in residual confounding. Fourth, ethnic classification relied on national identity card records without validation of three-generation family history, and inter-ethnic marriages may have led to misclassification, potentially underestimating the Zhuang population. Finally, due to questionnaire constraints, we were unable to differentiate between CKM stages 2 and 3, which may have obscured stage-specific association patterns. Future longitudinal studies are warranted to clarify causal pathways and temporal progression across CKM stages. More refined staging criteria and incorporation of genetic, lifestyle, and environmental data may further elucidate ethnic and regional heterogeneity. Additionally, interventional studies targeting central conditions such as hypertension could help validate network-informed prevention strategies.

## 5 Conclusion

This study demonstrated a high prevalence of CKM syndrome in Guangxi, a region in southern China where ethnic minority populations reside, highlighting the strong associations among CKD, CVD, and metabolic factors. We recommend multidisciplinary interventions within the CKM framework, focusing on health education, lifestyle modifications, chronic disease screening for high-risk populations, and the development of prediction models and novel therapies. Personalized care targeting multimorbidities is particularly crucial in economically underdeveloped and culturally distinct regions of China where minority populations reside.

## Data Availability

The original contributions presented in the study are included in the article/**Supplementary Material**. Further inquiries can be directed to the corresponding authors.
