# Supplementary material for: Prevalence of cardiovascular-kidney-metabolic syndrome in areas of Southern China where ethnic minority populations reside: a cross-sectional study
Source: Front Endocrinol (Lausanne). 2026 Apr 22;17:1818782. doi: 10.3389/fendo.2026.1818782 (PMC13143582; doi:10.3389/fendo.2026.1818782)
Supplement: Supplementary file 1 [file DataSheet1.docx]

Supplementary Material

Supplementary Table 1. Definitions of CKM syndrome stages

| **CKM syndrome stages** | **Definition** |
| --- | --- |
| **Stage 0:**  No CKM risk factors | Individuals with normal BMI and waist circumference, normoglycemia, normotension, a normal lipid profile, and no evidence of CKD or subclinical or clinical CVD |
| **Stage 1:**  Excess or dysfunctional adiposity | Individuals with overweight/obesity, abdominal obesity, or dysfunctional adipose tissue, without the presence of other metabolic risk factors or CKD BMI ≥24 kg/m^2^, Waist circumference ≥85/90 cm in women/men , or Fasting blood glucose 100–125 mg/dL or HbA1c between 5.7% and 6.4%. |
| **Stage 2:**  Metabolic risk factors and CKD | Individuals with metabolic risk factors (hypertriglyceridemia [≥135 mg/dL], hypertension, MetS, diabetes), or CKD |
| **Stage 3:**  Sub-clinical CVD in CKM | Subclinical ASCVD or subclinical HF among individuals with excess/dysfunctional adiposity, other metabolic risk factors, or CKD  Subclinical ASCVD to be principally diagnosed by coronary artery calcification (subclinical atherosclerosis by coronary catheterization/CT angiography also meets criteria)Subclinical HF diagnosed by elevated cardiac biomarkers (NT-proBNP ≥125 pg/mL, hs-troponin T ≥14 ng/L for women and ≥22 ng/L for men, hstroponin I ≥10 ng/L for women and ≥12 ng/L for men) or by echocardiographic parameters, with a combination of the 2 indicating highest HF risk.  Risk equivalents of subclinical CVD  Very high-risk CKD (stage G4 or G5 CKD or very high risk per KDIGO classification)  High predicted 10-y CVD risk |
| **Stage 4:**  Clinical CVD in CKM | Clinical CVD (coronary heart disease, HF, stroke, peripheral artery disease, atrial fibrillation) among individuals with excess/dysfunctional adiposity, other CKM risk factors, or CKD  Stage 4a: no kidney failure Stage 4b: kidney failure present |

Adapted from the AHA Scientific Statement on CKM Syndrome (Circulation, Nov 14, 2023; 148(20):1636-1664). Overweight/obesity and abdominal obesity criteria have been modified according to the standards for the Chinese population. Abbreviations: ASCVD indicates atherosclerotic cardiovascular disease; BMI, body mass index; CKD, chronic kidney disease; CKM, cardiovascular-kidney-metabolic; CT, computed tomography; CVD, cardiovascular disease; HbA1c, hemoglobin A1c; HDL, high-density lipoprotein; HF, heart failure; hs-troponin, high-sensitivity troponin; KDIGO, Kidney Disease Improving Global Outcomes; MetS, metabolic syndrome; and NT-proBNP; N-terminal pro-B-type natriuretic peptide.

**Supplementary Table 2. Characteristics of the study population**

| **Characteristic** | **Sex**  **n (%)** | | | **Residence**  **n (%)** | | | **Total**  **n (%)** |
| --- | --- | --- | --- | --- | --- | --- | --- |
|  | **Male**  **(n = 3587)** | **Female**  **(n = 4965)** | **p value** | **Urban**  **(n = 5696)** | **Rural**  **(n = 2856)** | **p value** | **(n = 8552)** |
| **Age** (y) |  |  |  |  |  |  |  |
| 18 to <45 | 1459 (40.7) | 1912 (38.5) | 0.083 | 2478 (43.5) | 893 (31.3) | <0.001 | 3371 (39.4) |
| 45 to ≤64 | 1301 (36.3) | 1906 (38.4) |  | 1937 (34.0) | 1270 (44.5) |  | 3207 (37.5) |
| ≥65 | 827 (23.1) | 1147 (23.1) |  | 1281 (22.5) | 693 (24.3) |  | 1974 (23.1) |
| **Ethnicity** |  |  |  |  |  |  |  |
| Zhuang | 1455 (40.6) | 2159 (43.5) | 0.007 | 2483 (43.6) | 1131 (39.6) | <0.001 | 3614 (42.3) |
| Other | 2132 (59.4) | 2806 (56.5) |  | 3213 (56.4) | 1725 (60.4) |  | 4938 (57.7) |
| **Educational Level** |  |  |  |  |  |  |  |
| primary education or below | 962 (26.8) | 2110 (42.5) | <0.001 | 1912 (33.6) | 1160 (40.6) | <0.001 | 3072 (35.9) |
| Middle school | 1995 (55.6) | 1904 (38.4) |  | 2705 (47.5) | 1194 (41.8) |  | 3899 (45.6) |
| College/University | 630 (17.6) | 951 (19.2) |  | 1079 (18.9) | 502 (17.6) |  | 1581 (18.5) |
| **Per capita income**  (last year, 10,000 CNY) |  |  |  |  |  |  |  |
| <2 | 799 (22.3) | 1160 (23.4) | 0.253 | 1200 (21.1) | 1026 (35.9) | <0.001 | 2226 (26.0) |
| 2 to <3 | 940 (26.2) | 1286 (25.9) |  | 1317 (23.1) | 642 (22.5) |  | 1959 (22.9) |
| 3 to <5 | 895 (25.0) | 1283 (25.8) |  | 1518 (26.7) | 660 (23.1) |  | 2178 (25.5) |
| ≥5 | 953 (26.6) | 1236 (24.9) |  | 1661 (29.2) | 528 (18.5) |  | 2189 (25.6) |
| **Smoking status** |  |  |  |  |  |  |  |
| Yes | 1553 (43.3) | 20 (0.4) | <0.001 | 1113 (19.5) | 460 (16.1) | <0.001 | 1573 (18.4) |
| No | 2028 (56.5) | 4936 (99.4) |  | 4576 (80.3) | 2388 (83.6) |  | 6964 (81.4) |
| **Alcohol consumption** |  |  |  |  |  |  |  |
| Yes | 1538 (42.9) | 191 (3.9) | <0.001 | 1172 (20.6) | 557 (19.5) | 0.258 | 1729 (20.2) |
| No | 2043 (57.0) | 4765 (96.0) |  | 4517 (79.3) | 2291 (80.2) |  | 6808 (79.6) |
| **Vegetable intake**  (<300 g/d) |  |  |  |  |  |  |  |
| Yes | 2295 (64.0) | 3052 (61.5) | 0.015 | 3337 (58.6) | 2010 (70.4) | <0.001 | 5347 (62.5) |
| No | 1286 (35.9) | 1910 (38.5) |  | 2355 (41.3) | 841 (29.4) |  | 3196 (37.4) |
| **Fruit intake**  (<200 g/d) |  |  |  |  |  |  |  |
| Yes | 2683 (74.8) | 3341 (67.3) | <0.001 | 4086 (71.7) | 1938 (67.9) | <0.001 | 6024 (70.4) |
| No | 898 (25.0) | 1621 (32.6) |  | 1610 (28.3) | 913 (32.0) |  | 2519 (29.5) |
| **Red meat intake**  (≥200 g/d) |  |  |  |  |  |  |  |
| Yes | 1424 (39.7) | 1816 (36.6) | 0.003 | 1997 (35.1) | 1243 (43.6) | <0.001 | 3240 (37.9) |
| No | 2157 (60.1) | 3146 (63.4) |  | 3695 (64.9) | 1608 (56.3) |  | 5303 (62.0) |
| **Physical inactivity** |  |  |  |  |  |  |  |
| Yes | 820 (22.9) | 1116 (22.5) | 0.688 | 1454 (25.5) | 482 (17.1) | <0.001 | 1936 (22.81) |
| No | 2742 (76.4) | 3811 (76.8) |  | 4217 (74.0) | 2336 (81.8) |  | 6553 (76.6) |
| **BMI (**kg/m²) |  |  |  |  |  |  |  |
| <18.5 | 198 (5.5) | 311 (6.3) | <0.001 | 384 (6.7) | 125 (4.4) | <0.001 | 509 (6.0) |
| 18.5 to <24 | 1613 (45.0) | 2543 (51.2) |  | 2818 (49.5) | 1338 (46.9) |  | 4156 (48.6) |
| 24 to <28 | 1276 (35.6) | 1568 (31.6) |  | 1849 (32.5) | 995 (34.8) |  | 2844 (33.3) |
| ≥28 | 500 (13.9) | 543 (10.9) |  | 645 (11.3) | 398 (13.9) |  | 1043 (12.2) |
| **Abdominal obesity** |  |  |  |  |  |  |  |
| Yes | 1011 (28.2) | 1317 (26.5) | 0.089 | 1519 (26.7) | 809 (28.3) | 0.104 | 2328 (27.2) |
| No | 2576 (71.8) | 3648 (73.5) |  | 4177 (73.3) | 2047 (71.7) |  | 6224 (72.8) |
| **Hypertension** |  |  |  |  |  |  |  |
| No hypertension | 2076 (57.9) | 3082 (62.1) | <0.001 | 3663 (64.3) | 1495 (52.4) | <0.001 | 5158 (60.3) |
| Newly diagnosed | 954 (26.6) | 1146 (23.1) |  | 1212 (21.3) | 888 (31.1) |  | 2100 (24.6) |
| Previously diagnosed | 557 (15.5) | 737 (14.8) |  | 821 (14.4) | 473 (16.6) |  | 1294 (15.1) |
| **Diabetes** |  |  |  |  |  |  |  |
| No diabetes | 1822 (50.8) | 2761 (55.6) | <0.001 | 3206 (56.3) | 1377 (48.2) | <0.001 | 4583 (53.6) |
| Prediabetes | 1260 (35.1) | 1673 (33.7) |  | 1869 (32.8) | 1064 (37.3) |  | 2933 (34.3) |
| Newly diagnosed | 380 (10.6) | 363 (7.3) |  | 431 (7.6) | 312 (10.9) |  | 743 (8.7) |
| Previously diagnosed | 125 (3.5) | 168 (3.4) |  | 190 (3.3) | 103 (3.6) |  | 293 (3.4) |
| **Dyslipidemia** |  |  |  |  |  |  |  |
| Yes | 1461 (40.7) | 1356 (27.3) | <0.001 | 1826 (32.1) | 991 (34.7) | 0.014 | 2817 (32.9) |
| No | 2126 (59.3) | 3609 (72.7) |  | 3870 (67.9) | 1865 (65.3) |  | 5735 (67.1) |
| **Hyperuricemia** |  |  |  |  |  |  |  |
| Yes | 1149 (32.0) | 844 (17.0) | <0.001 | 1364 (23.9) | 629 (22.0) | 0.047 | 1993 (23.3) |
| No | 2438 (68.0) | 4121 (83.0) |  | 4332 (76.1) | 2227 (78.0) |  | 6559 (76.7) |
| **History of kidney disease** |  |  |  |  |  |  |  |
| Yes | 7 (0.2) | 10 (0.2) | 0.949 | 14 (0.2) | 3 (0.1) | 0.168 | 17 (0.2) |
| No | 3580 (99.8) | 4955 (99.8) |  | 5682 (99.8) | 2853 (99.9) |  | 8535 (99.8) |
| **History of other CVD** |  |  |  |  |  |  |  |
| Yes | 83 (2.3) | 54 (1.1) | <0.001 | 74 (1.3) | 63 (2.2) | 0.002 | 137 (1.6) |
| No | 3504 (97.7) | 4911 (98.9) |  | 5622 (98.7) | 2793 (97.8) |  | 8415 (98.4) |
| **UACR ≥ 30 mg/g** |  |  |  |  |  |  |  |
| Yes | 351 (9.8) | 513 (10.3) | 0.408 | 532 (9.3) | 332 (11.6) | <0.001 | 864 (10.1) |
| No | 3236 (90.2) | 4452 (89.7) |  | 5164 (90.7) | 2524 (88.4) |  | 7688 (89.9) |
| **eGFR <60 ml/min/1.73m^2^** |  |  |  |  |  |  |  |
| Yes | 222 (6.2) | 221 (4.5) | <0.001 | 307 (5.4) | 136 (4.8) | 0.217 | 443 (5.2) |
| No | 3365 (93.8) | 4744 (95.6) |  | 5389 (94.6) | 2720 (95.2) |  | 8109 (94.8) |

The data of Smoking, drinking, Vegetable intake, fruit intake, red meat intake, Physical inactivity and history of other CVD was missing a few (<10%). Abbreviations: BMI, Body Mass Index; UACR, Urine Albumin-to-Creatinine Ratio; eGFR, Estimated Glomerular Filtration Rate. CVD, cardiovascular disease.

**
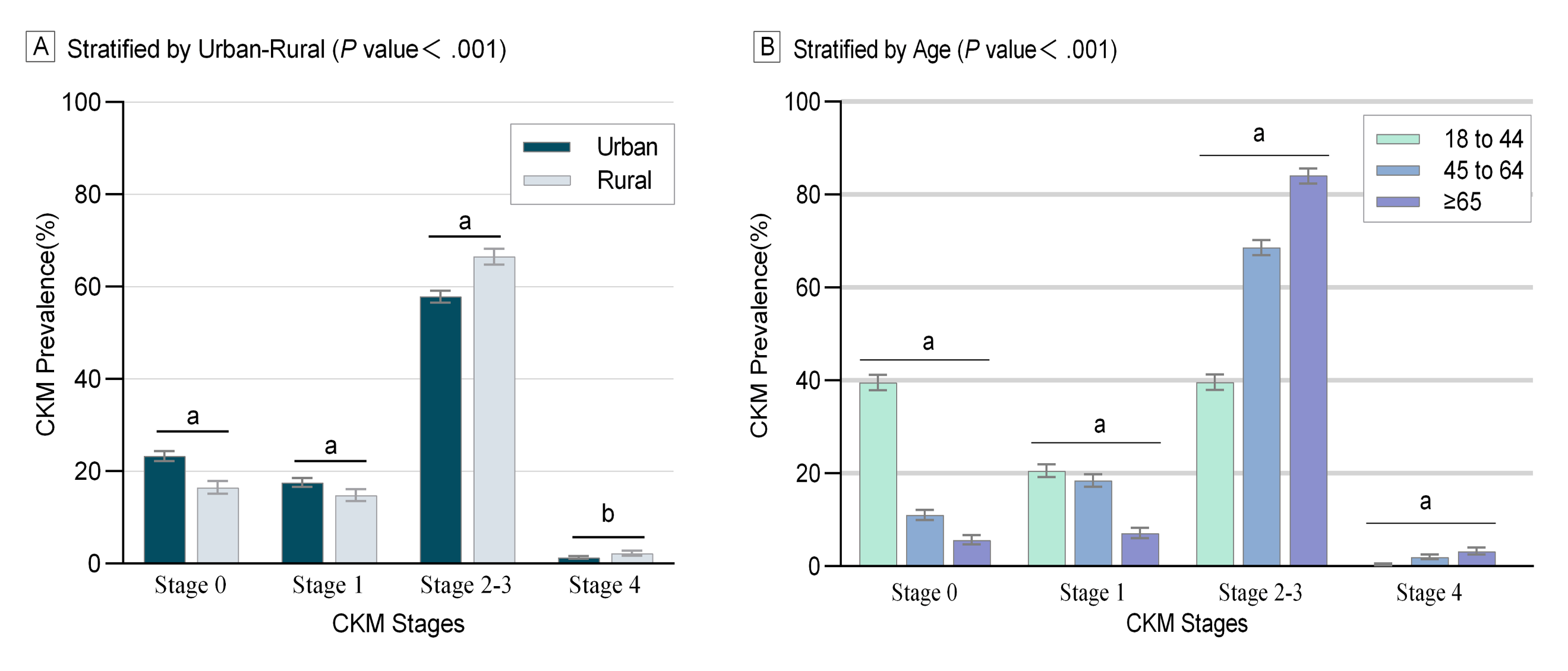
**

**Supplementary Figure 1. Prevalence of CKM stages in residence and age group.** Error bars indicate 95% confidence intervals. a Statistically significant between urban and rural groups (p < 0.001). b Statistically significant between age groups (*p* < 0.01).


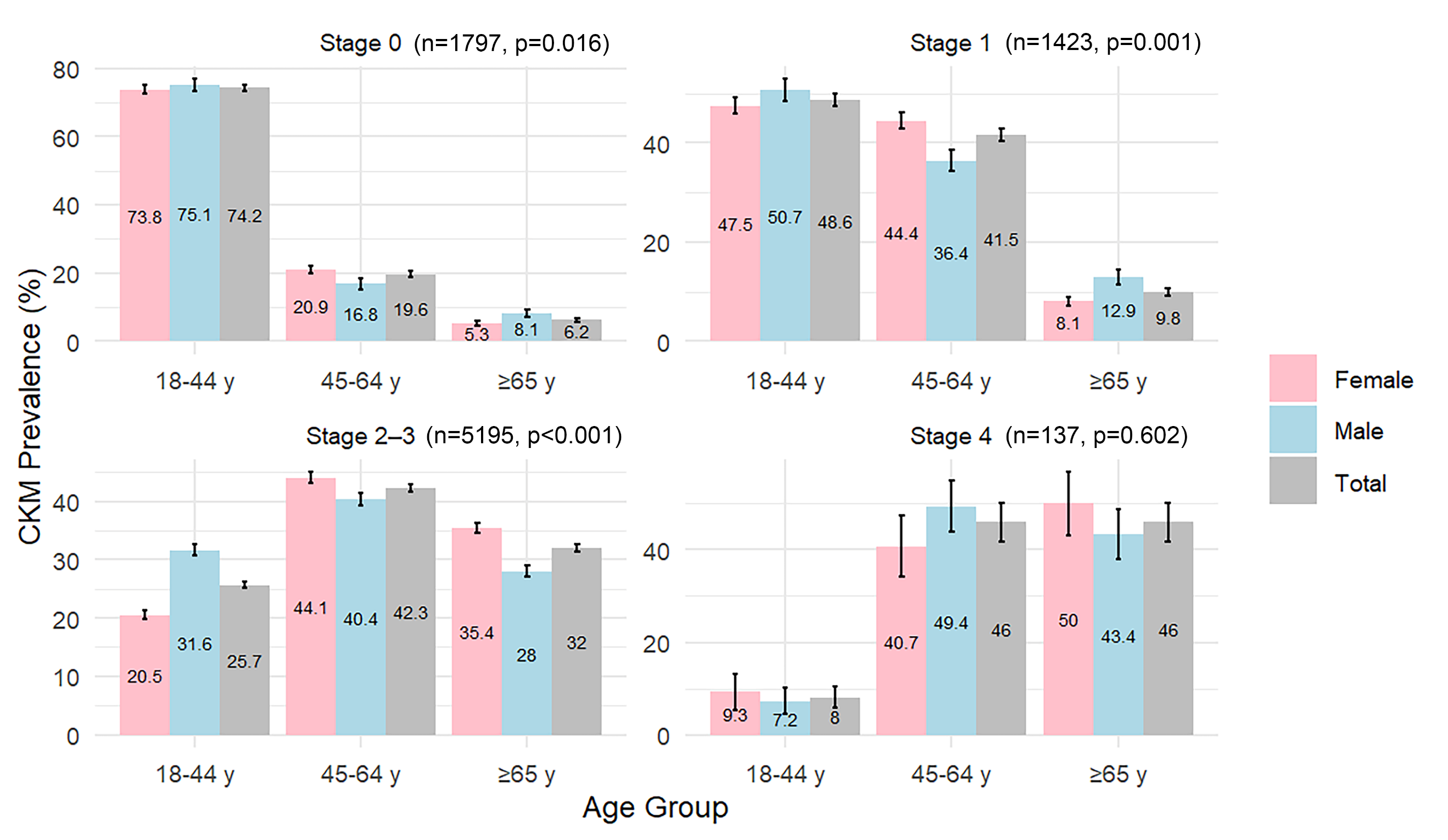


**Supplementary Figure 2. Prevalence of CKM stages in age and sex Group.** Error bars indicate 95% confidence intervals. P-values are all < 0.001. Abbreviations: CKM, Cardiovascular-Kidney-Metabolic.

**Supplementary Table 3. Multivariate binary logistic regression analysis of factors associated with CKM stages 1–4**

| **Characteristic** | **Univariate Analysis** | | **Multivariate Analysis** | |
| --- | --- | --- | --- | --- |
|  | ***OR (95%CI)*** | **P value** | ***OR (95%CI)*** | **P value** |
| **SEX** |  |  |  |  |
| Female | 1.00 (Reference) |  | 1.00 (Reference) |  |
| Male | 1.74 (1.56 ~ 1.94) | <0.001 | 1.62 (1.41-1.87) | <0.001 |
| **Age, y** |  |  |  |  |
| 18-44 | 1.00 (Reference) |  | 1.00 (Reference) |  |
| 45-64 | 5.29 (4.64 ~ 6.02) | <0.001 | 4.24 (3.69 - 4.89) | <0.001 |
| ≥65 | 10.98 (8.96 ~ 13.46) | <0.001 | 7.10 (5.71- 8.90) | <0.001 |
| **Ethnicity** |  |  |  |  |
| Zhuang | 1.00 (Reference) |  |  |  |
| Non-Zhuang | 1.01 (0.91 ~ 1.12) | 0.846 |  |  |
| **Educational Level** |  |  |  |  |
| College/University | 1.00 (Reference) |  | 1.00 (Reference) |  |
| Middle school | 2.07 (1.83 ~ 2.35) | <0.001 | 1.36 (1.19-1.57) | <0.001 |
| No or primary education | 6.29 (5.36 ~ 7.38) | <0.001 | 2.55 (2.12-3.07) | <0.001 |
| **Residence** |  |  |  |  |
| Urban | 1.00 (Reference) |  | 1.00 (Reference) |  |
| Rural | 1.54 (1.37 ~ 1.73) | <0.001 | 1.41 (1.24 - 1.61) | <0.001 |
| **Per capita income**  (last year, 10,000 CNY) |  |  |  |  |
| 2-<3 | 1.00 (Reference) |  |  |  |
| <2 | 1.38 (1.18 ~ 1.62) | <0.001 |  |  |
| 3-<5 | 0.87 (0.75 ~ 1.01) | 0.070 |  |  |
| ≥5 | 0.69 (0.60 ~ 0.80) | <0.001 |  |  |
| **Smoking status** |  |  |  |  |
| No | 1.00 (Reference) |  |  |  |
| Yes | 1.59 (1.37 ~ 1.85) | <0.001 |  |  |
| **Alcohol consumption** |  |  |  |  |
| No | 1.00 (Reference) |  | 1.00 (Reference) |  |
| Yes | 1.61 (1.40 ~ 1.86) | <0.001 | 1.38 (1.16-1.64) | <0.001 |
| **Vegetable intake**  (<300 g/d) |  |  |  |  |
| No | 1.00 (Reference) |  |  |  |
| Yes | 0.76 (0.68 ~ 0.85) | <0.001 | 0.81 (0.71 - 0.92) | <0.001 |
| **Fruit intake** (<200 g/d) |  |  |  |  |
| No | 1.00 (Reference) |  |  |  |
| Yes | 1.13 (1.01 ~ 1.27) | 0.031 |  |  |
| **Red meat intake**  ( ≥200 g/d) |  |  |  |  |
| No | 1.00 (Reference) |  | Reference |  |
| Yes | 1.25 (1.12 ~ 1.39) | <0.001 | 1.13 (0.99-1.28) | 0.062 |
| **Physical inactivity** |  |  |  |  |
| No | 1.00 (Reference) |  |  |  |
| Yes | 0.87 (0.77 ~ 0.99) | 0.030 |  |  |
| **Hyperuricemia** |  |  |  |  |
| No | 1.00 (Reference) |  | 1.00 (Reference) |  |
| Yes | 2.80 (2.39 ~ 3.26) | <0.001 | 2.70 (2.28- 3.20) | <0.001 |

Abbreviations: CKM, Cardiovascular-Kidney-Metabolic.


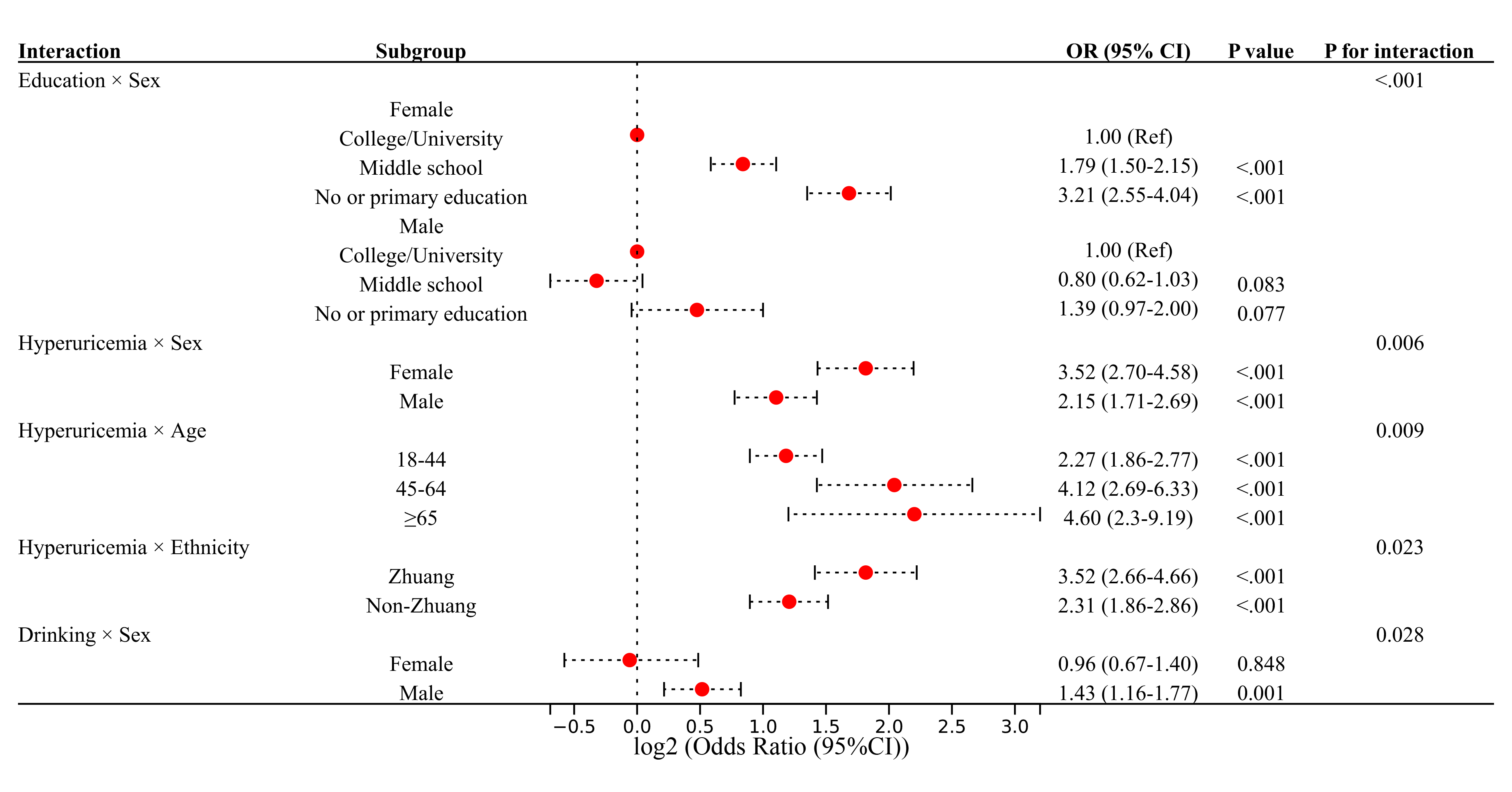


**Supplementary Figure 3. Stratified analysis of factors associated with CKM stages 1–4 by subgroups (P for interaction < 0.05).** Odds ratios (ORs) and 95% confidence intervals (CIs) are plotted on a log₂ scale. Each model included one interaction term between the stratification variable and the exposure of interest. All models were adjusted for gender, education level, urban/rural residence, age group, income, smoking status, fruit intake, vegetable intake, red meat intake, drinking status, exercise, and hyperuricemia. Abbreviations: OR, odds ratio; CI, confidence interval; CKM, cardiovascular–kidney–metabolic.

**Supplementary Table 4. Regression coefficients (log-odds ratios) for each edge in the CKM syndrome network**

|  | | **OVW** | | **OBE** | | **ABO** | | **DYS** | | **HTG** | | **MET** | | **HUA** | | **PDM** | | **DM** | | **HTN** | | **OCD** | | **IKF** | | **ALB** | | **CKD** | | **MKM** | | **EKM** | |
| --- | --- | --- | --- | --- | --- | --- | --- | --- | --- | --- | --- | --- | --- | --- | --- | --- | --- | --- | --- | --- | --- | --- | --- | --- | --- | --- | --- | --- | --- | --- | --- | --- | --- |
| **OVW** | | 0.000 | |  | |  | |  | |  | |  | |  | |  | |  | |  | |  | |  | |  | |  | |  | |  | |
| **OBE** | | -7.518 | | 0.000 | |  | |  | |  | |  | |  | |  | |  | |  | |  | |  | |  | |  | |  | |  | |
| **ABO** | | 1.224 | | 2.690 | | 0.000 | |  | |  | |  | |  | |  | |  | |  | |  | |  | |  | |  | |  | |  | |
| **DYS** | | 0.064 | | 0.000 | | 0.070 | | 0.000 | |  | |  | |  | |  | |  | |  | |  | |  | |  | |  | |  | |  | |
| **HTG** | | 0.196 | | 0.238 | | -0.844 | | 1.742 | | 0.000 | |  | |  | |  | |  | |  | |  | |  | |  | |  | |  | |  | |
| **MET** | | 0.240 | | 0.260 | | 2.707 | | 0.800 | | 2.506 | | 0.000 | |  | |  | |  | |  | |  | |  | |  | |  | |  | |  | |
| **HUA** | | 0.503 | | 0.931 | | 0.044 | | 0.167 | | 0.441 | | 0.193 | | 0.000 | |  | |  | |  | |  | |  | |  | |  | |  | |  | |
| **PDM** | | -0.575 | | -0.359 | | -0.146 | | 0.218 | | -0.312 | | 1.393 | | 0.071 | | 0.000 | |  | |  | |  | |  | |  | |  | |  | |  | |
| **DM** | | 0.081 | | 0.118 | | -0.479 | | 0.129 | | -0.975 | | 2.456 | | 0.000 | | -7.046 | | 0.000 | |  | |  | |  | |  | |  | |  | |  | |
| **HTN** | | 0.179 | | 0.196 | | -0.098 | | 0.108 | | -2.044 | | 1.085 | | 0.057 | | 0.285 | | -0.381 | | 0.000 | |  | |  | |  | |  | |  | |  | |
| **OCD** | | 0.000 | | 0.000 | | 0.000 | | 0.000 | | 0.000 | | 0.000 | | 0.218 | | 0.000 | | 0.000 | | 0.431 | | 0.000 | |  | |  | |  | |  | |  | |
| **IKF** | | 0.000 | | -0.561 | | 0.190 | | 0.000 | | 0.000 | | 0.000 | | 1.346 | | 0.000 | | 0.000 | | 0.431 | | 0.678 | | 0.000 | |  | |  | |  | |  | |
| **ALB** | | 0.000 | | 0.000 | | 0.000 | | 0.000 | | 0.000 | | 0.000 | | 0.000 | | 0.000 | | 0.000 | | 0.662 | | 0.000 | | -2.407 | | 0.000 | |  | |  | |  | |
| **CKD** | | -0.359 | | -0.340 | | 0.000 | | 0.000 | | -0.793 | | 0.322 | | 0.000 | | 0.146 | | 0.405 | | -0.476 | | 0.000 | | 9.190 | | 5.155 | | 0.000 | |  | |  | |
| **MKM** | | 1.464 | | 0.000 | | 2.686 | | 0.000 | | 10.656 | | 6.628 | | 0.000 | | 3.362 | | 8.950 | | 11.723 | | 6.200 | | 0.000 | | 0.000 | | 8.505 | | 0.000 | |  | |
| **EKM** | 4.968 | | 3.459 | | 6.700 | | 0.646 | | 0.000 | | 0.000 | | 0.000 | | 8.722 | | 0.000 | | 0.000 | | 0.000 | | 0.000 | | 0.000 | | 0.000 | | -14.883 | | 0.000 | |  |

Abbreviations: OVW, Overweight; OBE, Obesity; ABO, Abdominal Obesity; DYS, Dyslipidemia; HTG, Hypertriglyceridemia; MET, Metabolic Syndrome; HUA, Hyperuricemia; PDM, Prediabetes; DM, Diabetes; HTN, Hypertension; OCD,Other Cardiovascular Diseases; IKF, Impaired Kidney Function; ALB, Albuminuria; CKD, Chronic Kidney Disease; MKM, Moderate-to-advanced Cardiovascular-Kidney-Metabolic; EKM, Early Cardiovascular-Kidney-Metabolic.

**
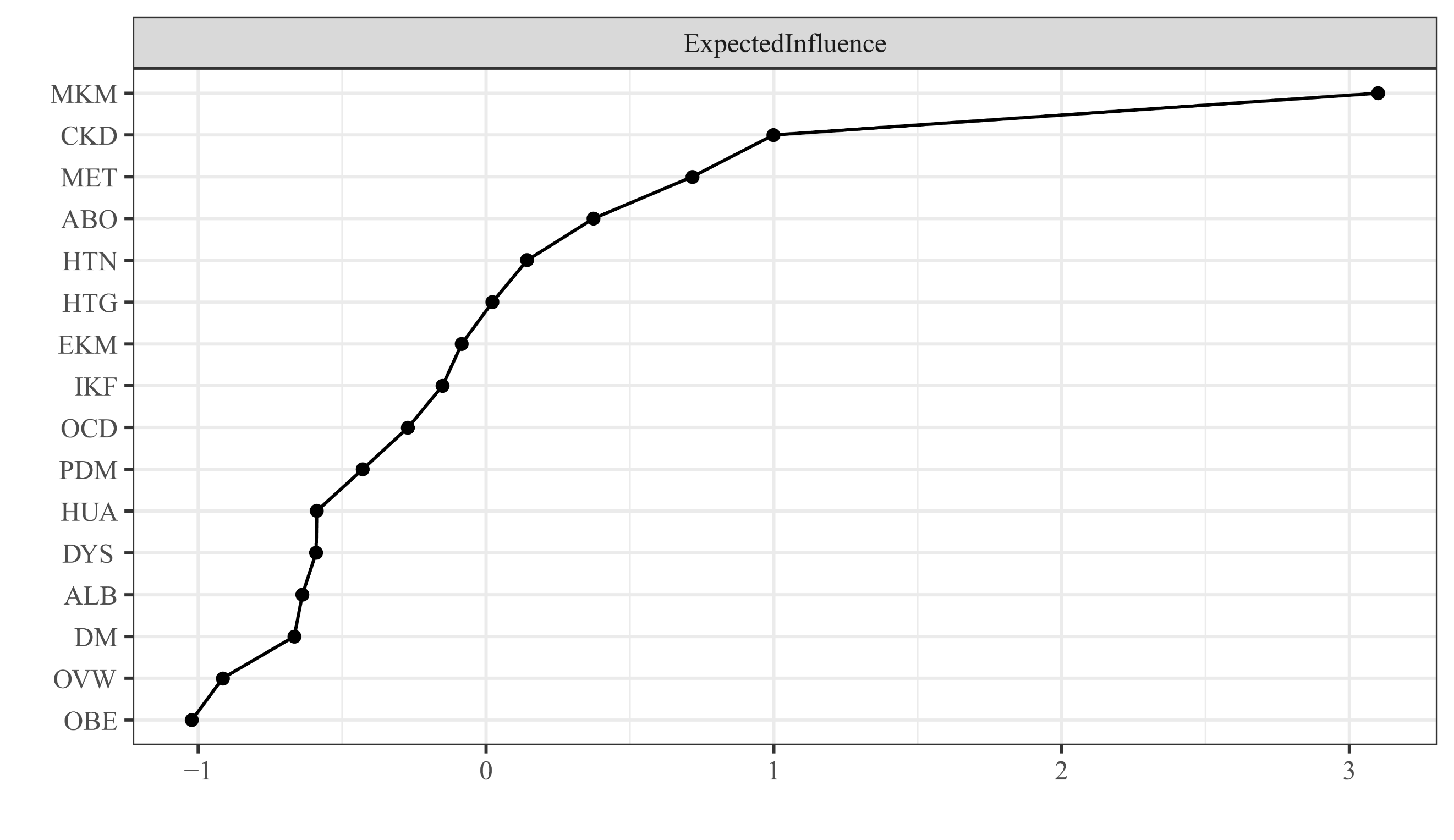
**

**Supplementary Figure 4. Expected influence centrality indices for CKM network structure.**

Abbreviations: OVW, Overweight; OBE, Obesity; ABO, Abdominal Obesity; DYS, Dyslipidemia; HTG, Hypertriglyceridemia; MET, Metabolic Syndrome; HUA, Hyperuricemia; PDM, Prediabetes; DM, Diabetes; HTN, Hypertension; OCD,Other Cardiovascular Diseases; IKF, Impaired Kidney Function; ALB, lbuminuria; CKD, Chronic Kidney Disease; MKM, Moderate-to-advanced Cardiovascular-Kidney-Metabolic; EKM, Early Cardiovascular-Kidney-Metabolic.


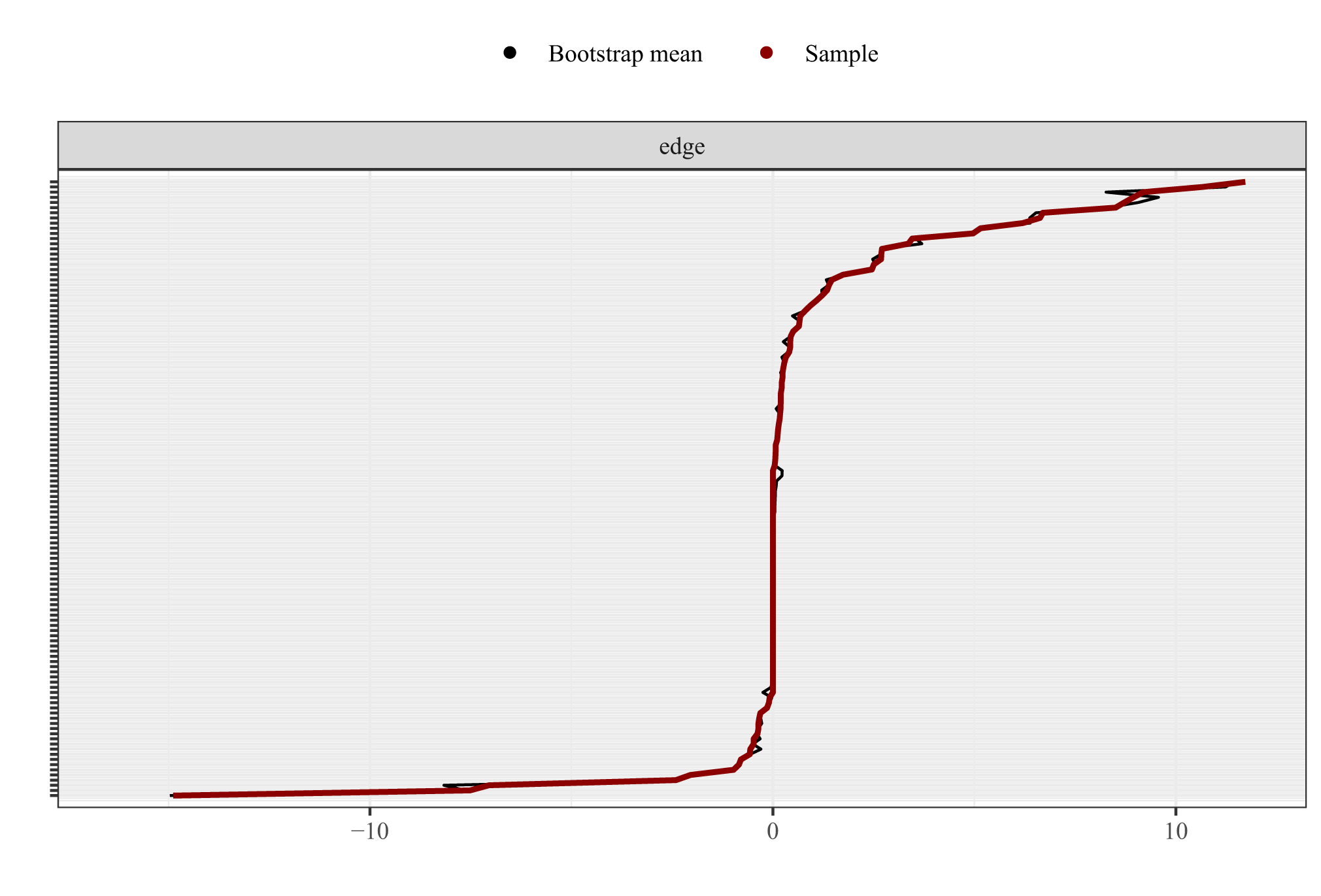


**Supplementary Figure 5. Stability analysis of CKM network edges.** The network demonstrated stable edge estimates. Bootstrap results (nBoots = 1000, nCores = 8) evaluated the accuracy of edge estimates through resampling. The gray area represents the 95% confidence intervals derived from resampling, the red line indicates the observed single-sample statistics, and the gray line shows the resampling mean. The alignment between the red and gray lines, with most edges falling within the confidence intervals, indicates stable edge estimates.


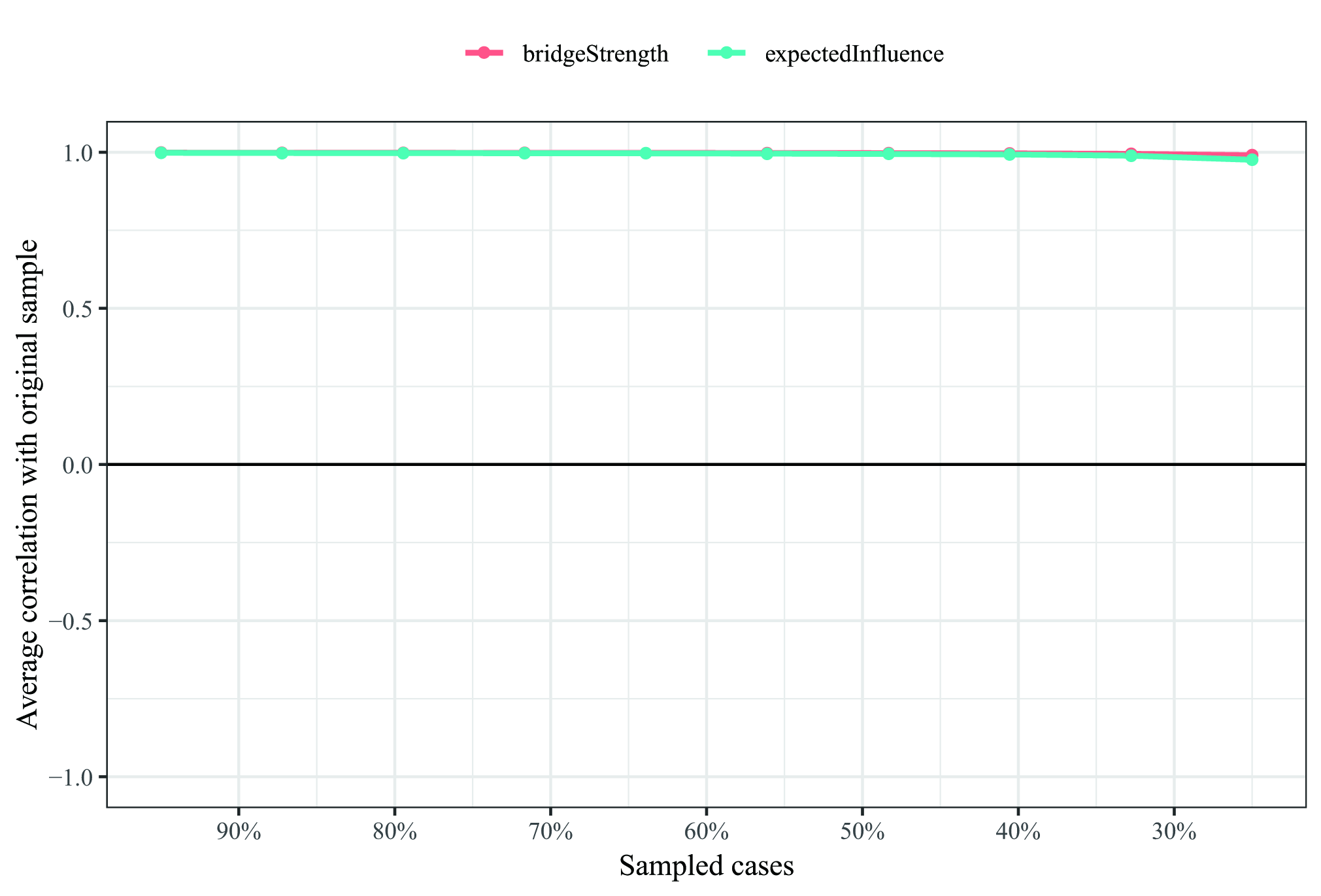


Supplementary Figure 6. Stability analysis of centrality indices in the CKM network. The stability of centrality indices was evaluated using bootstrapped subsampling (nBoots = 1000). The CS-coefficient (correlation stability coefficient) for expected influence and bridge strength centrality reached 0.75, indicating high stability when up to 75% of the data were removed.

**
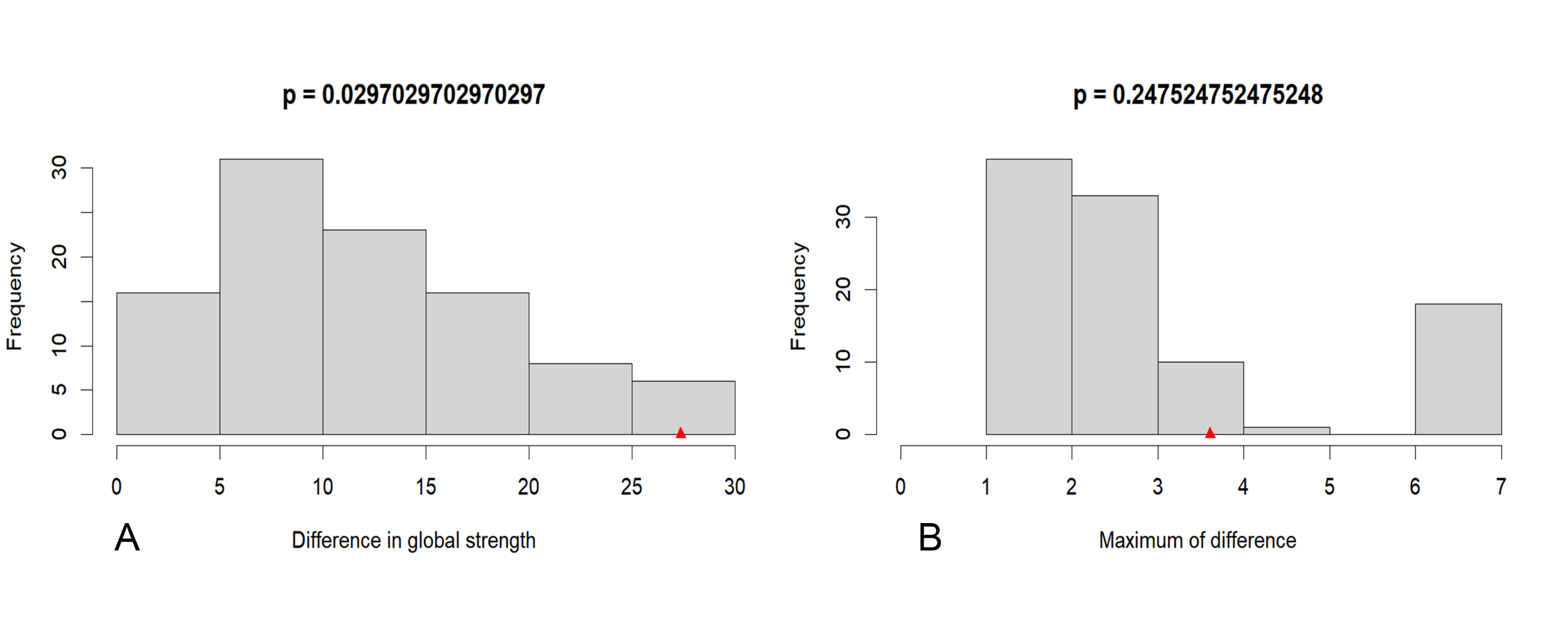
**

**Supplementary Figure 7. Comparison of network properties between urban and rural.** (A): A plot of bootstrap distribution of the difference in network global strength. The difference was significant (the global strength among the urban group: 165.3432; among the rural group: 137.9667; S = 27.37648, *p* = 0.02970297). (B): A plot of bootstrap distribution of the maximum difference in network structure between urban and rural areas (M = 3.608223, *p* = 0.2475248).
